# Impact of haematopoietic stem cell transplantation for benign and malignant haematologic and non-haematologic disorders on fertility: a systematic review and meta-analysis

**DOI:** 10.1038/s41409-025-02520-6

**Published:** 2025-02-26

**Authors:** Angela Vidal, Cristina Bora, Andrea Jarisch, Janna Pape, Susanna Weidlinger, Tanya Karrer, Michael von Wolff

**Affiliations:** 1 Division of Gynecological Endocrinology and Reproductive Medicine, Women’s University Hospital, Inselspital Bern, University of Bern, Bern, Switzerland; 2 Division of Pediatric Stem Cell Transplantation and Immunology, Department of Children and Adolescents, Frankfurt University Hospital, Goethe University Frankfurt, Frankfurt am Main, Germany; 3 Medical Library, University Library of Bern, University of Bern, Bern, Switzerland

**Keywords:** Haematological cancer, Haematological cancer

## Abstract

Haematopoietic stem cell transplantation (HSCT) exposes patients to long-term complications like gonadal dysfunction and infertility. The European Society for Blood and Marrow Transplantation advised in 2015 that fertility preservation should be considered for children and adolescents requiring HSCT. This systematic review and meta-analysis is part of the FertiTOX project, which aims to close the data gap regarding the gonadotoxicity of anticancer therapies to provide more accurate advice regarding fertility preservation. This review were conducted in November 2023, covering articles since 2000. In total, 56 studies were included in the meta-analysis, comprising 1853 female malignant, 241 female benign, 1871 male malignant, and 226 male benign cases. The analysis, using a random-effects model, estimated the prevalence and its 95% confidence interval, revealing that overall infertility exceeded 30% in all groups. Female malignant cases had a prevalence of 65% (95% CI: 0.58–0.71), while in females with benign disease, it was 61% (CI: 0.48–0.73). Males with malignant disease had a prevalence of 41% (CI: 0.32–0.51), and those with benign disease had 31% (CI: 0.19–0.46). The > 30% overall prevalence indicates a clinical need for fertility preservation counseling in both genders undergoing HSCT. Further prospective studies are necessary to address HSCT’s individual impact on gonadal function. This systematic review is registered with the International Prospective Register of Systematic Reviews (PROSPERO) under CRD42023486928.

## Introduction

Haematopoietic stem cell transplantation (HSCT) is a potentially curative or consolidative therapy which is widely used in patients with either malignant and benign disorders (Snowden et al. [1]; Saad et al. [2]). The most common indications for HSCT include haematological diseases (e.g., haematological malignancies such as leukemia, lymphoma, multiple myeloma) and haematological non-malignancies (e.g., aplastic anemia, thalassemia and Fanconi’s anemia), among other diseases [3, 4].

Due to the continuous expansion of HSCT indications, the number of HSCT continues to increase. In Europe in 2021, 47,412 HSCT were performed in 43,109 patients (19,806 (42%) allogeneic and 27,606 (58%) autologous). Simultaneously, 5-year survival rates after HSCT have improved, reaching 90% for some diseases [4, 5]. Consequently, there is increased awareness of various long-term complications such as infertility in patients receiving these highly effective therapies with high long-term survival. Previous reports show that the pregnancy rate after HSCT is less than 5% [6].

The knowledge regarding the toxicity of cancer treatments is expanding. Furthermore, treatment protocols are undergoing a transformation, resulting in enhanced outcomes, elevated survival rates, and a reduction in irreversible side effects. Given the high risk of primary ovarian insufficiency (POI) or testicular failure following HSCT conditioning and the negative impact of POI on fertility, it is imperative to consider protection of patients’ fertility prior to commencing HSCT.

Consequently, counselling about fertility preservation measures before the start of gonadotoxic therapies is crucial. However, fertility preservation in the context of diseases requiring HSTC is complex. For benign diseases in which HSTC is not urgently required or in which patients have not been pretreated with some kind of gonadotoxic therapies within a few month before the start of HSTC, all kind of fertility preservation measures such as freezing of oocytes, ovarian tissue, sperm and testicular tissue are feasible options. Conversely, in malignant diseases such as leukemia the situation is more complex. In males sperm can be frozen just before the start of the induction chemotherapy but in females time to freeze oocytes is too short. In some cases freezing of ovarian tissue can still be performed after induction chemotherapy and before HSTC has started if blood is free of leukemia cells. The situation becomes even more complex in case of prepubertal females and males in which only cryopreservation of ovarian and testicular tissue are feasible but experimental options in prepubertal cases [7–9].

Given the complexity of fertility preservation in patients definitely or possibly requiring HSTC, it is crucial to assess the expected and the potential risk of infertility before the onset of gonadotoxic therapies.

We therefore aimed to systematically assess the gonadal toxicity of myoablative treatments in both females and male patients with benign and malignant diseases. The study is part of the FertiTOX project (www.fertitox.com), which aims to close the data gap regarding the gonadotoxicity of anticancer therapies to provide more accurate advice about fertility preservation measures [10–14]. The intention of this systematic review and meta-analysis is to provide better guidance for oncologists and reproductive physicians regarding the risk of infertility and the necessity to consider fertility preservation measures. Even though HSTC treatments are heterogeneous which makes it difficult summarize these therapies, an overall estimation of the gonadotoxicity is urgently needed.

## Materials and methods

### Registration of protocols

This study protocol was registered in the Prospective International Registry of Systematic Reviews, (PROSPERO; registry number: CRD42023486928). The Preferred Reporting Criteria for Systematic Reviews and Meta-Analyses (PRISMA) were applied [15].

### Search strategy

**Fig. 1:**
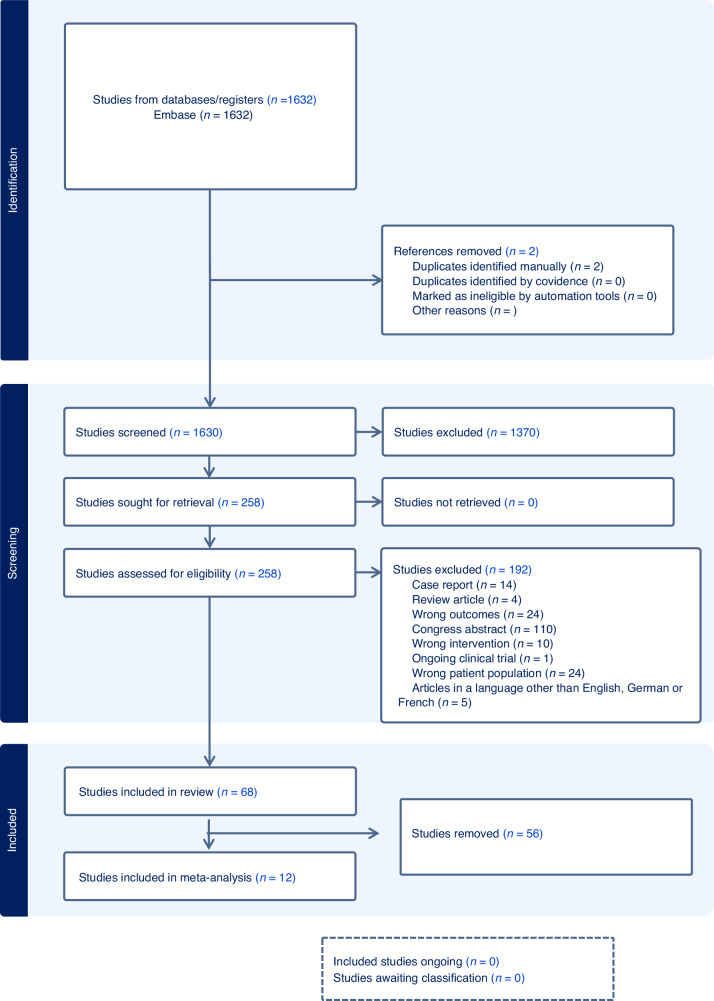
PRISMA flow diagram. Flowchart of the literature search and selection process.

We conducted a systematic literature search of the Medline, Embase, and Cochrane CENTRAL databases in November 2023 (Fig. 1). An initial MEDLINE search strategy was developed by a medical information specialist and tested using a list of basic references. After refining and querying, complex search strategies were established for each information source based on database-specific controlled vocabulary (i.e., thesaurus terms/subject headings) and text words. The text word search included synonyms, acronyms and similar terms. We limited our search to publications from 2000 to the present. Our search terms included all types of cancers for both benign and malignant haematologic diseases.

Animal-only studies were excluded from MEDLINE and Embase searches using a double- negative search strategy based on Ovid “humans-only” filters. Detailed final search strategies are presented in a supplementary file (S1). Reference lists and bibliographies of relevant publications were reviewed for relevant studies in addition to searching electronic databases. All of the identified citations were imported into Covidence. Duplicates entries were removed [16].

### Inclusion and exclusion criteria

Two investigators (AV and CB) independently assessed studies for inclusion using the Covidence software (www.covidence.org) [17]. All original papers with information on the type of haematologic disease, tumor therapy and fertility outcomes with numbers that allowed calculation of prevalence were eligible. The definitions of clinically relevant gonadal toxicity are described in Table 1. Studies that did not evaluate gonadal toxicity as defined in Table 1 were excluded.

**Table 1:** Clinically significant gonadal toxicity definitions. Definitions.

Females	Males
Menstrual cycle disorders - Amenorrhea/ Oligomenorrhea - Hormonal treatment: Puberty induction / Hormonal replacement therapy	Disorders of sperm quality - Azoospermia - Oligozoospermia
Hormone levels above the normal range - Follicle stimulating hormone (FSH) - Luteinizing hormone (LH)	Hormone levels above the normal range - Follicle stimulating hormone (FSH) - Luteinizing hormone (LH)
Premature ovarian insufficiency (POI) - oligo/amenorrhea for at least 4 months and - an elevated FSH level > 25 IU/l on two occasions at 4 weeks apart before the age of 40. - (*ESHRE Definition*) *	Gonadal dysfunction - Low testosterone levels - Hormonal treatment: Testosterone therapy
Low ovarian reserve parameters - Antimüllerian hormone (AMH)	Hormone levels below the normal range - Inhibin B

### Data extraction

Two investigators (AV and CB) summarized and independently reviewed the extracted data in detail (Tables 2, 3). The key variables of interest included the characteristics of the study populations (patient age at diagnosis and outcome; duration of follow-up, benign and malignant haematologic diseases, tumor class, type of oncological treatment, and fertility parameters).

**Table 2:** Characteristics of the included studies females.

First author, Year of publication	Country	Study design	Number of participants of interest (females)	Age of participants of interest at time of diagnosis/therapy (years, range)	Age (years, mean ± SD) at outcome/evaluation	Follow up after diagnosis/treatment, length in years (range)	Tumor type Number (%)	Chemotherapy, details	Radiotherapy, details	Suspected infertility	Comments
Afify et al. [39]	Australia	Retrospective longitudinal	8	10.88 (1.03–15.04)	Not specified	4.9 (2.01–9.98)	AML	By + Cy	Not specified	7/8 (87.5%)*	*Calculated in women with hypergonadotropic hypogonadism
Bakker et al. [43]	The Nether Lands	Retrospective longitudinal	19	11.3 (0.9–15.9)	19 (16.1–25.4)	Min. 5 years	AML ALL CML MDS NHL	*Conditioning regimen:* Cy, ARA-C, VP16	TBI Testicular irradiation or cranial irradiation	13/19 (68%)*	*Calculated in women with hypergonadotropic hypogonadism after a minimum of 5 years of follow-up
Maeda et al. [106]	Japan	Retrospective	42	11 (5–16)	17 (13–27)	5 (3–14y)	SAA 12 AML 13 ALL 12 CML 3 NHL 2	Bu + MEL	TBI	28/42 (67%)*	*Calculated in women with hypergonadotropic hypogonadism
Tauchmanova et al. 2003	Italy	Retrospective	45	29 (21–45)	Not specified	2	AML19 CML 7 ALL 4 CLL 3 HL 8 NHL 4	Bu + Cy BEAM *GVHD prophylaxis:* CsA + MTX	Not specified	39/45 (87%)*	*Calculated in women with hypergonadotropic hypogonadism
Li et al. [73]	China	Retrospective	10	8.8 (1.8–18.8)	14.3 (6.7–25.2)	5.5 (2–8.8)	TM	Bu + Cy + ATG *GVHD prophylaxis:* CsA + MTX	Not specified	10/10(100%)*	*Calculated in women with hypergonadotropic hypogonadism
Somali et al. [74]	Greece	Retrospective	41	30.4 (9.7)	32.6 (10.0)	1.5 (0.2–9.8)	ALL 9 CML 13 AML 21 NHL 4 HL 16 OT 9	Bu + Cy BECYM (carmustine, VP16, Cy, MEL) BEAM	No	40/41 (97%)*	*Calculated in women with hypergonadotropic hypogonadism
Vlachopapadopoulou et al. [75]	Greece	Retrospective	13	15.2 (9.5–18.9)	Not specified	Not specified	β -TM	Bu + Cy *GVHD prophylaxis:* CsA + MTX + ATG	Not specified	12/13 (92.3%)*	*Calculated in women with hypergonadotropic hypogonadism
Claessens et al. [76]	The Nether Lands	Retrospective	27	36 (14–54)	Not specified	Not specified	ALL 24 ALL 13 CML 33	Not specified	Not specified	26/27 (96%)*	*Calculated in women with hypergonadotropic hypogonadism
Couto-Silva et al. [77]	France	Retrospective	34	7.2 ± 0.6 (0.6–13.1)	14.3 ± 0.4 (10.1–19.7)	10	AML HL ALL FA SAA CID OT	Not specified	TBI	22/34 (64.7%)*	*Calculated in women with hypergonadotropic hypogonadism
Shalitin et al. [78]	Israel	Retrospective	39	7.4 ± 5.2 (0.6–21.5)	Not specified	6.27 ± 3.5 (1–22.5)	ALL 10 AML 22 CML 4 HL 8 Neuroblastoma 16 Sarcoma 4 Medulloblastoma 2 WT 1 Germ cell tumor 1 HB 1 AA 5 FA 4 β-TM 9 Wiskott-Aldrich syndrome 2 Hemophagocytic syndrome 2	Irradiation + Cy and/or other drugs (Bu, MEL, VP-16 or ATG) 18 Cy and/or Bu, MEL, carboplatin, THIO or VP-16 or ATG *GVHD prophylaxis:* MTX + CsA	TBI 14 TAI 3	24/39(62%)*	*Calculated in women with hypergonadotropic hypogonadism
Brachet et al. [35]	Belgium	Retrospective longitudinal	10	9.4 (2.2–14.2)	15.5 (12–26)	Not specified	SCD	Bu + Cy 18 *GVHD prophylaxis:* CsA + MTX	No	7/10 (70%)*	*Calculated in women with hypergonadotropic hypogonadism and need for puberty induction
Lie Fong et al. [44]	The Nether Lands	Retrospective	12	25.3 (17.9–36.6)	31.4 (22.2–40.6)	Not specified	Not specified (malignant)	Cy, procarbazine and/or ifosfamide 10 MTX or idarubicin 3 TBI + Cy 12	TBI	11/12 (92%)*	*Calculated in women with hypergonadotropic hypogonadism, amenorrhea rate and undetectable AMH
Steffens et al. [40]	Belgium	Retrospective	6	10.0 (2.9–13.9)	26.9 (17.0-28.9)	15.6 ± 2.9	ALL 78 NHL 16	MEL + Cy + TBI 18	TBI	6/6 (100%)*	*Calculated in women with hypergonadotropic hypogonadism
Nabhan et al. [79]	Japan	Retrospective	12	12 (5–19)	Not specified	14 (9-20)	FA	Cy + ovarian irradiation Cy only 3 *GVHD prophylaxis:* CsA + MTX	TBI or TAI	5/12 (41.6%)	*Calculated in women with hypergonadotropic hypogonadism
Cho et al. [80]	Korea	Retrospective	50	9.9 (SD± 3.4)	15.0 (SD± 2.2)	12	ALL 13 AML 25 CML 2 SAA 8 FA 1 OT 1	Bu + TBI 4 Bu 19 RIC 4	TBI 23/50	36/50 (72%)*	*Calculated in women with hypergonadotropic hypogonadism
Felicetti et al. [22]	Italy	Prospective	16	10.3 (3.1–23.2)	24.7 (18.8–34.3)	13.1 (7.1–23.4)	ALL AML CML	Acridinyl anisidine, ATG, Bu, carboplatin, carmustine, Cy, ARA-C, VP16, FLUD, MEL, THIO, vincristine	TBI 23	10/16 (62.5%)*	*Calculated in women with hypergonadotropic hypogonadism
Ho et al. [81]	Canada	Retrospective	41	8.74 (0.79–17.94)	11.84 (1.13–21.17)	1	ALL 21 Myelogenous 20 Solid tumors 21 HL 5 NHL 2 MDS 2 Benign 8 Histiocytosis 5 Hurler’s syndrome 1	Not specified	Not specified	11/41 (26.8%)*	*Calculated in women with hypergonadotropic hypogonadism
Laporte et al. [61]	France	Retrospective	34	7.0 ± 0.6 (0.6–13)	13.9 ± 0.3 (11–17.3)	8.3 ± 0.6	ALL 31 AML 16 CML 3 Lymphoma 5 Neuroblastoma 5 Nephroblastoma 1 AA 6 CID 4 Myelodysplasia 1	Cy, MEL and/or Bu ARA-C 12 VP16 5(400 mg/m^2^) MTX 4 Vincristine 5	TBI or TLI	27/34 (79%)*	*Calculated in women with hypergonadotropic hypogonadism
Borgmann-Staudt et al. [31]	Germany	Retrospective	133	13 (4–28)	19 (12–35)	6 (3–12)	ALL 114 (33%) AML 42 (12%) CML 31 (9%) MDS 30 (9%) SAA 29 (8%) ALD 23 (7%) FA 12 (4%) ES 10 (3%) TM 7 (2%) NHL 5 (2%) OT 41 (12%)	Bu/Cy 64 (33%) Bu/Cy/MEL 36 (18%) Cy 22 (11%) Others 74 (38%) TBI/VP16 95 (64%) TBI/Cy 28 (19%) TBI/others 25 (17%)	TBI 148 (43%)	111/133 (83%)*	*Calculated in women with hypergonadotropic hypogonadism
Khalil et al. [28]	Israel	Retrospective	27 (20)	7.7 (1.1–32)	Not specified	12	β-TM 43 SCD 4	Bu + Cy Protocol P26 (hydroxyurea, azathioprine, Bu, Cy) Protocol P26.1 (+ THIO and ATG) *GVHD prophylaxis:* CsA + MTX	Not specified	16/20 (80%)*	*Calculated in women with hypergonadotropic hypogonadism on the mature females
Blijdorp et al. [32]	The Nether Lands	Retrospective	5	11.5 (1.1–15.0)	32.4 (23.4–44.5)	19.0 (11.6–30.0)	AML MDS CML	Not specified (with Cy)	TBI	5/5 (100%)*	*Calculated in women with AMH below the value of reference.
Inagaki et al. [36]	Japan	Retrospective	5	10.3 (2–19)	12	10 (1.8–26.4)	SAA 18 Hypocellular 5	Cy ± ATG	TBI	0-5%	*Calculated in women with hypergonadotropic hypogonadism and spontaneous menstruation
Krawczuk-Rybak et al. [33]	Poland	Retrospective	6	15.2 ± 2.6	21.35 ± 4.4	Not specified	Leukemia	4 × MVPP + 4 × B-DOPA: Dacarbazine Procarbazine Nitrogen mustard Bu, Cy	TBI 1	6/6 (100%)*	*Calculated in women with hypergonadotropic hypogonadism and lower AMH
Poomthavorn et al. [82]	Thailand	Retrospective	22	7.6 (1.0-18.7)	Not specified	Not specified	β -TM	Bu + Cy 36 ± FLUD + ATG RIC (Bu, FLUD, ATG) 11 *GVHD prophylaxis:* CsA + MTX	TLI 2	17/22 (77%)*	*Calculated in women with hypergonadotropic hypogonadism
Bresters et al [51]	The Nether Lands	Retrospective	109	Pre-pubertal: 6.5 (0.3–12.3) Pubertal: 13.5 (11.2–14.6) Post-pubertal: 15.0 (11.9–18.9)	Pre-pubertal:19.6 (10.9–36.3) Pubertal: 27.2 (15.8–41.8) Post-pubertal: 26.0 (15.3–45.2)	7.7	AML ALL MDS CML NHL CID AA β-TM FA Blackfan-Diamond anemia	Cy 39/44 VP16 5 Bu 34 MEL 13 Ifosfamide 2	TBI 53%	61/109 (56%)* -Chemotherapy with Bu: 23 (68%) -Chemotherapy without Bu: 5 (29%) -TBI/TAI: 33 (57%)	*Calculated in women with Ovarian insufficiency
Pup et al. [83]	Italy	Retrospective	17	26 (18–37)	Not specified	Not specified	NHL 9 HL 8	CHOP ± rituximab Second line DHAOX 2 Third-line CDE 1 ABVD 3 VEPEB 3 MOPP/ ABV 2 Second line IGEV 8	TAI 9 Ovarian irradiation 3	7/17 (41%)*	*Calculated in women not recovery menstruation
Vatanen et al. [26]	Sweden, Finland	Retrospective longitudinal	92	9 ± 4.3 (1–19)	22 ± 6.3 (9–41)	13 ± 5.5 (6–27)	ALL 33 AML 24 SAA 13 Others 22	Cy / MEL + TBI ± VP16 Cy + Bu	F/s TBI TLI	55/92 (60%)*	*Calculated in women with hypergonadotropic hypogonadism
Elchuri et al. [37]	USA	Retrospective cross-sectional cohort	9	8.9 (3.3–12.9)	Not specified	5.5 (1.0–9.7)	SCD	Bu + Cy	Not specified	9/9 (100)* 8/9 (89%)**	*Calculated in women with undetectable AMH **Calculated in women with hypergonadotropic hypogonadism
Pfitzer et al. [107]	Germany	Retrospective longitudinal	144	13.3 (3.7–28.4)	22.9 (12.5–39.2)	Not specified	ALL 113 (31 %) AML 41 (11 %) SAA 29 (8 %) CML 28 (8 %) MDS 28 (8 %) ALD 22 (6 %) FA 12 (3 %) ES 7 (2 %) β-TM 2 (1 %) NHL 4 (1 %) OT 75 (21 %)	Bu, carboplatin, cisplatin, Cy, VP16, ifosfamide, MEL and procarbazin	TBI Pelvic irradiation	82/144 (57%)*	*Calculated in women with hypergonadotropic hypogonadism
Barnum et al. [84]	USA	Retrospective	15	10.7 (3.3–34.3)	13.8 (5.9–36.3)	Not specified	FA 44	TBI + Cy, FLUD ± ATG *GVHD prophylaxis:* Methylprednisolone, MMF, CsA or sirolimus	TBI	6 /15 (40%)*	*Calculated in women with hypergonadotropic hypogonadism
Kawano et al. [52]	Japan	Retrospective	69	12.5 (1–37)	Not specified	Not specified	Not specified	Bu + at least one other alkylator (Cy) TBI + Cy ± ARA-C TBI + Cy + VP-16 TBI + MEL RIC (FLUD + MEL) Cy ± ATG	TBI	40/69 (58%)*	*Calculated in women with hypergonadotropic hypogonadism
Nakano et al. [85]	Japan	Retrospective	11	21 (19–31)	Not specified	Not specified	ALL 7 SAA 4	Cy + TBI ARA-C + TBI 1 RIC (FLUD, Cy, ATG) 2 RIC (FLUD, MEL, alemtuzumab + TBI) 1 *GVHD prophylaxis:* Calcineurin inhibitor + MTX	TBI	11/11 (100%)*	*Calculated in women with undetectable AMH
Vaezi et al. [70]	Israel	Retrospective	36	Not specified	Not specified	1	AML 72 ALL 50	Bu/Cy	Not specified	15%*	*Calculated in women with hypergonadotropic hypogonadism
Gokcebay et al. [46]	Turkey	Retrospective	41	8.8 ± 5.0	12.2 ± 6.2	3.4 ± 1.2 (1–5.5)	AML 12 ALL 19 MDS 4 CML 2 Solid tumor 9 β-TM 29 AA 15 OT 3	Cy A *GVHD prophylaxis:* MTX *Conditioning regimen:* Bu 62 Bu-Cy (+VP16, +Mel, +ATG) 33 Pesaro protocol (26) 12 Bu-Flu-ATG 3	TBI 20 -TBI-VP16 (+Cy) 12 (5) -TBI-Cy 3	14/41(34.1%)*	*Calculated in women with hypergonadotropic hypogonadism
Rahal et al. [24]	France	Retrospective	54	5.9 (3.1–11.2)	20 (14.2–28.3)	11.9 (7–19.3)	β -TM	Bu + Cy + ATG 67 *GVHD prophylaxis:* CsA + MTX 58	Not specified	26/54 (58%)*	*Calculated in women with hypogonadism
Abou-Mourad et al. [86]	Canada	Retrospective	92	39 (14–57)	Not specified	8.6 (2.3–22.8)	CML117 (27.2%) AML 112 (26.1%) NHL 53 (12.3%) MDS/myelofibrosis 40 (9.3%) MM 39 (9.1%) ALL 34 (7.9%) SAA/PNH 17 (4.0%) CLL 10 (2.3%) Others 7 (1.6%)	*Conditioning regimen:* Bu/Cy ± other agent(s) 227 (52.9%) Others (chemotherapy-only) 9 (2.1%) *GVHD prophylaxis:* CsA + MTX ± other drug(s) 378 (88.1%) CsA ± other drug(s) 29 (6.8%) MTX ± other drug(s) 8 (1.8%) T-cell depletion ± other drug(s) 6 (1.4%)	TBI 193 (45%)	68.8%*	*Calculated in women with hypergonadotropic hypogonadism after a minimum of 2-5 years of follow-up
Ashizawa et al. [87]	USA	Retrospective longitudinal	15 (19)	23 (19–33)	Not specified	3.9 (0.17–10.12)	AML 9 (47%) ALL 5 (26%) MDS 2 (10%) AUL 1 (5%) BPDCN 1 (5%) SAA 1(5%)	*Conditioning regimen:* Cy, ARA-C *GVHD prophylaxis:* Csa/tacrolimus + MTX	TBI	3/15 (15%)*	*Calculated in women who need HRT
Faraci et al. [88]	Europe	Retrospective	27	11.04 (5.02–18.55; IQR, 7.61–15.01)	19.39 (8.23–33.86) [16.1–23.53]	7.79 (2.07–22.69) [6.2–10.51]	CID Hemophagocytic lymphohistiocytosis CML JMML	Bu or treosulfan	Not specified	-prepubertal 14/27 (51.8%)* -postpubertal 13/21 (70%)*	*Calculated in women with administered HRT
Komori et al. [89]	Japan	Retrospective	19 (14)	9.6 (0.8–22.8)	Not specified	12.2 (3.7–15.7)	ALL 4 AML 6 MDS 1 JMML 1 OT 2	FLUD, Cy	TBI	3/14 (21.4)*	*Calculated in women with hypergonadotropic hypogonadism
Weinhard et al. [90]	France	Retrospective longitudinal	28	7 (0–13)	19 (15–47)	Not specified	ALL (37.8%) AML (17.6%) Other malignancy (14.9%) Benign (29.7%)	Bu	TBI	85.80%	*Calculated in women with partial or complete hypogonadism
Bhatt N et al. [91]	USA	Prospective	32	9.7 (5.8)	30.4 (7.6)	Not specified	AML	Not specified	TBI 45 (68.2%)	14/32 (43.8%)*	*Calculated in women with hypergonadotropic hypogonadism
Boga et al. [92]	Turkey	Prospective observational	22	29 (18–45)	33 (20–50)	2	SCD	Not specified	Not specified	10/22 (45%)*	*Calculated in women with hypergonadotropic hypogonadism
Elchuri et al. [23]	USA	Retrospective	24	9 (1.8–16.7)	7.9 (± 5.6)	13	SCD	Cy + Bu Or only Bu	Not specified	17/24 (71%)*	*Calculated in women with hypergonadotropic hypogonadism
Felicetti et al. [21]	Italy	Retrospective	40	0–4y 30 (22.9%) 5–9y 25 (19.08%) ≥ 10y 76 (58.02%)	24.68 (21.47–28.27)	Not specified	NHL HL AML MDS	Alkylating agents and/or Cy	Not specified	34/40(85%)*	*Calculated in women with hypergonadotropic hypogonadism
Shimazaki et al. [93]	Japan	Retrospective	36	4.3 (0–15.6)	17.4 (7.1–35.3)	8.0 (0.33–21.6)	Brain tumors 11 Solid tumors 11 ALL 22 AML12 NHL 8 SAA 3 Wiskott-Aldrich syndrome 1 JMML 1	Alkylating agents, anthracyclines, methotrexates, heavy metals and asparaginase RIC (Cy + ATG) 1	TBI TLI 4	14/36 (38%)*	*Calculated in women with hypergonadotropic hypogonadism
Forgeard et al. [94]	France	Retrospective longitudinal	58*/34**	23.4 (18.3–28)	30.9 (24.2–36.9)	2	ALL HL MDS Glanzmann disease Hemoglobinopathy	Alkylating agents and/or conditioning regimen (MAC, RIC)	TBI 10	57/58 (98%)* 25/34(73%)**	*Calculated in women with administered HRT **Calculated in women with hypergonadotropic hypogonadism
Molinari et al. [95]	Italy	Retrospective	43	12.3–33.1	Not specified	2	Leukemia 58 Lymphoma 58 Benign 8	Not specified	Abdominopelvic irradiation	31/43 (72.1%)	*Calculated in women with hypergonadotropic hypogonadism
Wikander et al. [27]	Sweden	Prospective	39	0–19	Not specified	17 (0–31)	ALL 5 AML 7 Other leukemia 5 Lymphoma 4 Sarcoma 2 Neuroblastoma 2 AA 5 Thalassemia 3 OT 6	Bu, MEL or other alkylating chemotherapy	TBI	18/35 (51.4%)*	*Calculated in women with hypergonadotropic hypogonadism
Xue et al. [96]	China	Retrospective longitudinal	25	31 (18–66)	Not specified	2 (0–8)	AML 25 ALL 17 NHL 9 HL 1 AA 8 MM 6 MDS 2	Bu/FLUD Mbu/Ara-C Bu/Cy+VP16 Bu/Cy FLUD/Cy TBI/Cy Bu/Cy+VP16 High-dose MEL BEAM *GVHD prophylaxis:* CsA, MMF, MTX	TBI	23/25 (92%)*	*Calculated in women with low AMH
George et al. [97]	Georgia	Retrospective	17	7.5 (3.7–20.3)	Not specified	2	SCD	Not specified (CEDs were calculated)	TBI (Regimen E)	15/17(88.2%)*	*Calculated in women with undetectable AMH
Guemes et al. [48]	Spain	Retrospective	62	8.0 (± 5.2)	Not specified	3.5 (0.5–10.6)	ALL 42 AML 34 Lymphomas 5 Congenital anemias 13 Cytopeni 12 Solid tumors 40 OT (rare entities) 11	FLUD + Bu + THIO Bu + MEL Cy + FLUD + thymoglobulin FLUD + Bu *GVHD prophylaxis:* CsA ± glucocorticoids, thymoglobulin	TBI 8	28/62 (45.7%)*	*Calculated in women with hypergonadotropic hypogonadism
Su et al. [98]	China	Cross-sectional	55	10.9–47.3	Not specified	Not specified	ALL 21 (38.2%) AML 23 (41.8%) AA 5 (9.1%) OT 6 (10.9%)	TBI + Cy 45.5% Modified Bu/Cy regimen 41.8%	TBI 45.5%	40/55 (72.7%)*	*Calculated in women with hypergonadotropic hypogonadism
Chabut et al. [99]	France	Prospective	178	7.7 (4.79–10.90)	24.9 (21.08–30.19)	18 (14.2–23.3)	ALL AML	Bu 62 Bu + Cy 49 Bu +MEL 6 Bu + MEL + aracytine 5 Bu + FLUD + THIO 2 TBI + Cy 53 TBI + Cy + FLUD 2 TBI + MEL 3 TBI + ARA-C + MEL 50 TBI + VP16 8	TBI 116	139/178 (78%)	*Calculated in women with hypergonadotropic hypogonadism
Jin et al. [20]	Korea	Retrospective	16	12.4 ± 4.4 (0.4–19.8)	24.0 ± 4.7 (15.2–33.8)	10.8 (1–16.5)	Leukemia 18 (26.1%) Lymphoma 4 (5.8%) CNS tumor 6 (8.7%) Embryonal tumor 1 (1.5%) Bone/soft tissue sarcoma 19 (27.5%) Ovarian tumor 21 (30.4%)	Not specified	21 (30.4%) Pelvis or TBI 9 (13.0%)	11/16 (78.6%)*	*Calculated in women with hypergonadotropic hypogonadism
Koo et al. [19]	USA	Retrospective	57	8.2 (4.8–24.9)	Not specified	7.7 (0.1–24.7)	FA	Bu-based regimen	Low dose irradiation	30/57 (52.6%)	*Calculated in women with hypergonadotropic hypogonadism

Summary of cohort studies assessing the prevalence of gonadal toxicity in women.

The studies are sorted by year of publication. Age and duration of follow-up are given as years with mean (SD), or range where such data are available.

Abbreviations:

Diagnosis: *AA* aplastic anemia, *AML* acute myeloid leukemia, *AUL* acute unclassified leukemia, *ALL* acute lymphoblastic leukemia, *ALD* Adrenoleukodystrophy, *BPDCN* blastic plasmacytoid dendritic cell neoplasm, *β-TM* β-thalassemia major, *CID* congenital immunodeficiency, *CML* Chronic myeloid leukemia, *CLL* Chronic lymphocytic leukemia, *CNS* central nervous system, *FA* Fanconi Anemia, *HB* hepatoblastoma, *HL* Hodgkin’s lymphoma, *JMML* Juvenile Myelomonocytic Leukemia, *MDS* Myelodysplastic syndrome/myelofibrosis, *MM* Multiple myeloma, *NHL* non-Hodgkin’s lymphoma, *OT* Others, *SAA* Severe aplastic anemia/PNH, *SCD* Sickle Cell Disease, *WT* Wilms tumor.

Chemotherapy: *ABVD* doxorubicin, bleomycin, vinblastine and dacarbazine, *ARA-C* cytarabine, *ATG* anti-thymocyte globulin, *BEAM* carmustine, *VP16* cytarabine, MEL, *Bu* busulfan, *CDE* doxorubicin, cyclophosphamide, etoposide, and dexamethasone, *CHOP* cyclophosphamide, doxorubicin, vincristine, and prednisolone, *CsA* cyclosporine A, *Cy* cyclophosphamide, *DHAOX* oxaliplatin, cytarabine, and dexamethasone, *FLUD* fludarabine, *IGEV* ifosphamide, gemcitabine, and vinorelbine, *MEL* melphalan, *MMF* mycophenolate mofetil, *MOPP/ABV(D)* mechlorethamine, vincristine, procarbazine, prednisone, doxorubicin, bleomycin, vinblastine, (dacarbazine), *THIO* thiotepa, *VEPEB* cyclophosphamide, epirubicin, vinorelbine, bleomycin, and prednisolone.

Radiotherapy: *TAI* thoracoabdominal irradiation, *TBI* total body irradiation, *TLI* total lymphoid irradiation.

Parameters: *AMH* anti-Mullerian hormone.

**Table 3:** Characteristics of the included studies males.

First author, year of publication	Country	Study design	Number of participants of interest (males)	Age of participants of interest at time of diagnosis/therapy (years, range)	Age (years, mean ± SD) at outcome/evaluation	Follow up after diagnosis/treatment, length in years (range)	Tumor type Number (%)	Chemotherapy, details	Radiotherapy, details	Infertility	Comments
Afify et al. [39]	Australia	Retrospective longitudinal	15	10.88 (1.03–15.04)	Not specified	4.9 (2.01–9.98)	AML (100))	Bu + Cy	Not specified	0/14 (0%*)	*Calculated in men with testosterone levels below normal range with the need for testosterone replacement therapy.
Bakker et al. [43]	The Nether lands	Retrospective longitudinal	21	11.3 (0.9–15.9)	19 (16.1–25.4)	Min. 5 years	AML ALL CML MDS NHL	Cy Cy + ARA-C VP16	TBI	0/19 (0%*)	*Calculated in men with low testosterone levels after a minimum of 5 years of follow-up. The 2 males who underwent radiotherapy to the testes were excluded.
Chatterjee et al. [66]	UK	Retrospective	117	34 (18–68)	Not specified	3–18 months	NHL 35 HL 36 AML 17 ALL 11 MM 12 CML 6	Multi-agent chemotherapy *GVHD prophylaxis:* CsA + MTX	TBI 34	15/117 (12.8%)	*Calculated in men with hypergonadotropic hypogonadism
Schimmer et al. [67]	Canada	Retrospective	16	36 (20–49)	39 (24–50)	3 (0–8)	NHL 3 HL 9 AML 4	VP16 + MEL ABVD 5 MOPP/ABV 1 MOPP/ABVD 1 ProMACE-CytaBOM 1 VACOP-B 1 Mini-BEAM 8 DHAP 3	TBI	6/16 (37.5%*)	*Calculated in men with hypergonadotropic hypogonadism
Anserini et al. [71]	Italy	Retrospective	64	27.39 (9–50)	Not specified	4 (1–18)	SAA 11(17.1%) ALL 7 (10.9%) AML 25 (39%) CML 17 (26%) NHL 3(4.6%) HL 1 (%)	Cy Cy + TBI or TAI Cy + THIO Cy + Bu	TBI or TAI	45/64 (70.3%*)	*Calculated in men with azoospermia
Li et al. [73]	China	Retrospective	10	8.8 (1.8–18.8)	14.3 (6.7–25.2)	5.5 (2–8.8)	TM	Bu + Cy + ATG *GVHD prophylaxis:* CsA + MTX	Not specified	2/10 (20%)	*Calculated in men with hypergonadotropic hypogonadism and required hormonal replacement
Somali et al. [74]	Greece	Retrospective	31	30.4 (9.7)	32.6 (10.0)	1.5 (0.2–9.8)	ALL 9 CML 13 AML 21 NHL 4 HL 16 OT 9	Bu + Cy BECYM (carmustine, VP16, Cy, MEL BEAM	No	6/31 (19%*)	*Calculated in men with hypogonadism
Vlachopapadopoulou et al. [75]	Greece	Retrospective	12	13.5 (4.9–18.7)	Not specified	Not specified	β-TM	Bu + Cy *GVHD prophylaxis:* CsA + MTX + anti-lymphocyte globulin	Not specified	2/12 (16.6%*)	*Calculated in men with hypergonadotropic hypogonadism
Claessens et al. [76]	The Nether lands	Retrospective	15	32 (16–54)	Not specified	Not specified	ALL 24 ALL 13 CML 33	Not specified	Not specified	13/15 (87%*)	*Calculated in men with azoospermia
Couto-Silva et al. [77]	France	Retrospective	41	8.1 ± 0.5 (2–13.6)	14.7 ± 0.3 (11–20.6)	10	Neuroblastoma AML HL ALL FA SAA CID Nephroblastoma Neuroepithelioma	Not specified	TBI or TLI	25/41 (61%*)	*Calculated in men with tubular failure
Shalitin et al. [78]	Israel	Retrospective	52	7.4 ± 5.2 (0.6–21.5)	Not specified	6.27 ± 3.5 (1–22.5)	ALL 10 AML 22 CML 4 HL 8 Neuroblastoma 16 Sarcoma 4 Medulloblastoma 2 WT 1 Germ cell tumor 1 HB 1 AA 5 FA 4 β-TM 9 Wiskott-Aldrich syndrome 2 Hemophagocytic syndrome 2	Irradiation + Cy and/or other drugs (Bu, MEL, VP-16 or ATG) 18 Cy and/or Bu, MEL, carboplatin, THIO or VP-16 or ATG *GVHD prophylaxis:* MTX + CsA	TBI 14 TAI 3	9/52 (29%*)	*Calculated in men with hypogonadism
Brachet et al. [35]	Belgium	Retrospective longitudinal	9	9.4 (2.2–14.2)	15.5 (12 - 26)	Not specified	SCD	Bu + Cy 18 *GVHD prophylaxis:* Csa + MTX	No	9/9 (100%)	*Calculated in men with developed germinal epithelial failure(small testicular volume along with elevated FSH) during or after puberty.
Steffens et al. [40]	Belgium	Retrospective	12	10.0 (2.9–13.9)	26.9 (17.0–28.9)	15.6 ± 2.9	ALL 78 NHL 16	MEL + Cy + TBI 18	TBI	10/12 (83%)*	*Calculated in men with hypogonadism
Lukusa et al. [42]	Belgium	Retrospective	6	15.5 (8–21)	23 (18–34)	Not specified	SCD	Cy + Bu Cy + Bu + TLI *GVHD prophylaxis:* CsA + MTX	TLI	3/6 (50%*)	*Calculated in men with azoospermia
Abou-Mourad et al. [86]	Canada	Retrospective	42	39 (14–57)	Not specified	8.6 (2.3–22.8)	CML117 (27.2%) AML 112 (26.1%) NHL 53 (12.3%) MDS/myelofibrosis 40 (9.3%) MM 39 (9.1%) ALL 34 (7.9%) SAA/PNH 17 (4.0%) CLL 10 (2.3%) OT 7 (1.6%)	*Conditioning regimen:* Bu/Cy±other agent(s) 227 (52.9%) Others (chemotherapy-only) 9 (2.1%) *GVHD prophylaxis* CsA+MTX±other drug(s) 378 (88.1%) CsA±other drug(s) 29 (6.8%) MTX±other drug(s) 8 (1.8%) T-cell depletion±other drug(s) 6 (1.4%)	TBI 193 (45%)	8.5%*	*Calculated in men with testosterone levels below normal range with the need for testosterone replacement therapy.
Felicetti et al. [22]	Italy	Prospective	24	10.3 (3.1–23.2)	24.7 (18.8–34.3)	13.1 (7.1–23.4)	ALL AML CML	Acridinyl anisidine, ATG, Bu, carboplatin, carmustine, Cy, ARA-C, VP16, FLUD, MEL, THIO, vincristine	TBI 23	3/24 (12.5%*)	*Calculated in men with hypergonadotropic hypogonadism, all in the group with TBI
Ho et al. [81]	Canada	Retrospective	45	8.74 (0.79–17.94)	11.84 (1.13–21.17)	1	ALL 21 Myelogenous 20 Solid tumors 21 HL 5 NHL 2 MDS 2 Benign 8 Histiocytosis 5 Hurler’s syndrome 1	Not specified	Not specified	8/44 (18%*)	*Calculated in men with hypergonadotropic hypogonadism
Laporte et al. [61]	France	Retrospective	38	8.2 ± 0.6 (1.0–15)	16.5 ± 0.3 (13.2–21.3)	8.3 ± 0.6	ALL 31 AML 16 CML 3 Lymphoma 5 Neuroblastoma 5 Nephroblastoma 1 AA 6 CID 4 Myelodysplasia 1	Cy, MEL and/or Bu ARA-C 12 VP16 5(400 mg/m2) MTX 4 Vincristine 5	TBI or TLI	28/38 (74%*)	*Calculated in men with hypergonadotropic hypogonadism
Borgmann-Staudt et al. [31]	Germany	Retrospective	190	13 (4–28)	19 (12–35)	6 (range 3–12)	ALL 114 (33%) AML 42 (12%) CML 31 (9%) MDS 30 (9%) SAA 29 (8%) ALD 23 (7%) FA 12 (4%) ES 10 (3%) TM 7 (2%) NHL 5 (2%) OT 41 (12%)	Bu/Cy 64 (33%) Bu/Cy/MEL 36 (18%) Cy 22 (11%) Others 74 (38%) TBI/VP16 95 (64%) TBI/Cy 28 (19%) TBI/others 25 (17%)	TBI 148 (43%)	131/190 (69%*)	*Calculated in men with hypogonadism or testosterone substitution or in men with azoospermia
Khalil et al. [28]	Israel	Retrospective	19	7.7 (1.1–32)	Not specified	12	β-TM 43 SCD 4	Bu + Cy Protocol P26 (hydroxyurea, azathioprine, Bu, Cy) Protocol P26.1 (+ THIO and ATG) *GVHD prophylaxis:* CsA + MTX	Not specified	4/11 (36%*)	*Calculated in men with hypergonadotropic hypogonadism mature males
Blijdorp et al. [32]	The Nether lands	Retrospective	4	11.5 (1.1–15.0)	32.4 (23.4–44.5)	19.0 (11.6–30.0)	AML MDS CML	Not specified (with Cy)	TBI	4/4 (100%*)	*Calculated in men with very low Inhibin values as well as low testicular volume.
Inagaki et al. [36]	Japan	Retrospective	13	10.3 (2-19)	12	10 (1.8–26.4)	SAA 18 hypocellular 5	CY ± ATG	TBI	2/13 (15.3%*)	*Calculated in men with hypergonadotropic hypogonadism
Poomthavorn et al. [82]	Thailand	Retrospective	25	7.6 (1.0–18.7)	Not specified	Not specified	β-TM	Bu + Cy 36 ± FLUD + ATG RIC (Bu, FLUD, ATG) 11 *GVHD prophylaxis:* CsA + MTX	TLI 2	12/25 (48%*)	*Calculated in men with hypergonadotropic hypogonadism
Wilhelmsson et al. [26]	Sweden, Finland	Retrospective longitudinal	106	8 ± 4.6 (1–17)	22 ± 6.0 (12–42)	13 ± 4.8 (4–28)	ALL 20 AML 44 SAA 17 OT 25	Cy or cytarabine or MEL + TBI ± VP16 Cy + Bu	TBI	21/31 (67.7%*) 28/106 (26.4%**)	*Calculated in men with azoospermia **Calculated in men who need induction of substitution therapy.
Pfitzer et al. [107]	Germany	Retrospective longitudinal	217	13.3 (3.7–28.4)	22.9 (12.5–39.2)	Not specified	ALL 113 (31 %) AML 41 (11 %) SAA 29 (8 %) CML 28 (8 %) MDS 28 (8 %) ALD 22 (6 %) FA 12 (3 %) ES 7 (2 %) TM 2 (1 %) NHL 4 (1 %) OT 75 (21 %)	Bu, carboplatin, cisplatin, Cy, VP16, ifosfamide, MEL and procarbazin	TBI Pelvic irradiation	123/217 (56 %*)	*Calculated in men with hypogonadism or testosterone substitution or in men with azoospermia
Barnum et al. [84]	USA	Retrospective	15	10.7 (3.3–34.3)	13.8 (5.9–36.3)	Not specified	FA 44	TBI + Cy, FLUD ± ATG *GVHD prophylaxis:* Methylprednisolone, MMF, csa or sirolimus	TBI	2/15 (13.3%*)	*Calculated in men with low testosterone levels
Vaezi et al. [70]	Israel	Retrospective	58	Not specified	Not specified	1	AML 72 ALL 50	Bu/Cy	Not specified	49/58 (84%*)	*Calculated in men with hypogonadism and receiving testosterone replacement
Gokcebay et al. [46]	Turkey	Retrospective	59	8.9 ± 4.7	12.3 ± 5.9	3.4 ± 1.2 (1–5.5)	AML 12 ALL 19 MDS 4 CML 2 Solid tumors 9 β-TM 29 AA 15 OT 3	Cy A *GVHD prophylaxis:* MTX *Conditioning regimen:* Bu 62 Bu-Cy (+VP16, +Mel, +ATG) 33 Pesaro protocol (26) 12 Bu-Flu-ATG 3	TBI 20 TBI + VP16 (+Cy) 12 (5) TBI + Cy 3	1/37 (1.6%)	*Calculated in men with hypergonadotropic hypogonadism
Rahal et al. [24]	France	Retrospective	45	5.9 (3.1–11.2)	20 (14.2–28.3)	11.9 (7–19.3)	β-TM	Bu + Cy + ATG 67 *GVHD prophylaxis:* CsA + MTX 58	Not specified	3/45 (6.6%)	*Calculated in men with hypogonadism/delayed puberty
Duca et al. [38]	Italy	Prospective	5	6.6 SD ± 5.2	27.0 SD ± 5.7	Not specified	ALL AML HL NHL OT WT HB	Not specified	Not specified	5/5 (100%*)	*Calculated in men with azoospermia
Faraci et al. [88]	Europe	Retrospective	5	11.04 (5.02–18.55; IQR, 7.61–15.01).	19.39 (8.23–33.86) [16.1–23.53]	7.79 (2.07–22.69) [6.2–10.51]	CID Hemophagocytic lymphohistiocytosis CML JMML	Bu or treosulfan	Not specified	5/5 (100%*)	*Calculated in men with azoospermia
Taneja et al. [105]	UK	Retrospective	42	3.7 (0.7–14.1)	29.6 (14.5–47.7)	19.4 (5.0–33.1)	Childhood leukemia (whilst pre‐ or peripubertal)	MRC UKALL VIII‐XI, MRC 97 and 2003 regimens R1‐R314-16 regimen for relapses AML X protocol	TBI 24 (57%) Testicular boost 14 (33%) Additional cranial radiation 12 (28%)	37/42 (62%*)	*Calculated in men with hypogonadism and receiving testosterone replacement
Weinhard et al. [90]	France	Retrospective longitudinal	46	7 (0–13)	19 (15–47)	Not specified	ALL (37.8%) AML (17.6%) Other malignancy (14.9%) Benign (29.7%)	Bu	TBI	69.90%	*Calculated in men with partial or complete hypogonadism
Zhao et al. [34]	USA	Retrospective longitudinal	5	15 (11–19)	20 (18–25)	5 (3–11)	SAA SCD X‐linked lymphoproliferative disease	Alemtuzumab 4 + FLUD + MEL + ATG + Cy	TBI	5/5 (100%)	*Calculated in men with oligozoospermia / azoospermia
Bhatt N et al. [91]	USA	Prospective	34	9.7 (5.8)	30.4 (7.6)	Not specified	AML	Not specified	TBI 45	21/34 (64.7%*) 14/21 (67%**)	*Calculated in men with primary hypogonadism **Calculated in men with azoospermia
Boga et al. [92]	Turkey	Prospective	21	29 (18–45)	33 (20–50)	2	SCD	Not specified	Not specified	14/21 (74%*)	*Calculated in men with azoospermia
Elchuri et al. [23]	USA	Retrospective	16	11 (5.4–19.9)	7.9 (± 5.6)	13	SCD	Cy +/- Bu	Not specified	0/16 (0%)*	*Calculated in men with very low testosterone and high LH levels
Felicetti et al. [21]	Italy	Retrospective	50	0–4y 37 (18.88%) 5–9y 55 (28.06%) ≥ 10y 104 (53.06%)	24.57 (21.84–29.39)	Not specified	NHL HL AML MDS	Alkylating agents and/or Cy	Not specified	17/50 (34%*)	*Calculated in men with hypergonadotropic hypogonadism/Leydig cell failure
Mathiesen et al. [30]	Denmark	Cross-sectional	98	9.7 (0.4–16.9)	28.1 (18.5–47.0)	18.3 (7.7–34.6)	ALL 45 AML 9 MDS 9 NHL 5 Other leukemia (biphenotypic leukemia, CML, JMML) 6· SAA 13 CID 6 Benign 5	Bu + Cy (+ MEL/VP16/THIO) TBI + Cy TBI + Cy/Bu-Cy/VP16	TBI	15/71 (21%*) 30/98 (30%**)	*Calculated in men who need induction of substitution therapy. **Calculated in men with hypergonadotropic hypogonadism and azoospermia
Rostami et al. [104]	Iran	Cross-sectional	43	27.31 ± 4.34 (16–41)	Not specified	Not specified	β-TM	Bu + Cy *GVHD prophylaxis:* CsA + MTX 58	No	15/43 (36.6%*) 22/45 (48.8%**)	*Calculated in men with hypogonadism / **azoospermia
Shimazaki et al. [93]	Japan	Retrospective	33	4.3 (0–15.6)	17.4 (7.1–35.3)	8.0 (0.33–21.6)	Brain tumors 11 Solid tumors 11 ALL 22 AML12 NHL 8 SAA 3 Wiskott-Aldrich syndrome 1 JMML 1	Alkylating agents, anthracyclines, methotrexates, heavy metals and asparaginase RIC (Cy + ATG) 1	TBI TLI 4	7/33 (21%*)	*Calculated in men with hypogonadism
Lopez et al. [103]	France	Retrospective	188	8.8 ± 5.1	Not specified	Not specified	Childhood and adolescent leukemia	Bu	TBI (155/178, 87.1%)	105/188 (55%*)	*Calculated in men with hypergonadotropic hypogonadism
Mathiesen et al. [102]	Denmark	Cross-sectional	39	9.4 (0.4–16.9)	27.6 (18.5–40.4)	Not specified	AML 12 AML 6 MDS 5 NHL 4 Other leukemia (biphenotypic, JMML, CML) SAA 5 CID 2 benign 3	Bu + Cy (+ MEL/VP16/THIO) TBI + Cy TBI + Cy/Bu-Cy/VP16	TBI	prepubertal at HSCT: 12/26 (46%*) postpubertal at HSCT: 11/13 (84%*)	*Calculated in men with spontaneous puberty and developed hypergonadotropic hypogonadism and azoospermia
Xue et al. [96]	China	Restrospective longitudinal	47	31 (18–66)	Not specified	2 (0–8)	AML 25 ALL 17 NHL 9 HL 1 AA 8 MM 6 MDS 2	Bu/FLUD Mbu/Ara-C Bu/Cy+VP16 Bu/Cy FLUD/Cy TBI/Cy Bu/Cy+VP16 High-dose MEL BEAM *GVHD prophylaxis:* CsA, MMF, MTX	TBI	29/47 (62%)	*Calculated in men with hypergonadotropic hypogonadism
Zavattaro et al. [101]	Italy	Cross-sectional	44	49.08 (39.92–58.20)	53.25 (44.11–62.43)	3.68 (1.59–5.90)	AML ALL MM MDS NHL HL Blastic plasmacytoid dendritic cell neoplasm SAA	MAC regimen 87	TBI 27 Testicular boost 3	12/44 (27.27%)*	*Calculated in men with hypergonadotropic hypogonadism, after a minimum of 1-3 years of follow-up
Guemes et al. [48]	Spain	Retrospective	95	8.0 (5.2)	Not specified	3.5 (0.5–10.6)	ALL 42 AML 34 Lymphomas 5 Congenital anemias 13 Cytopenias 12 Solid tumors 40 OT (rare entities) 11	FLUD + Bu + THIO Bu + MEL Cy + FLUD + thymoglobulin FLUD + Bu *GVHD prophylaxis:* CsA ± glucocorticoids, thymoglobulin	TBI 8	25/95 (26.9%*)	*Calculated in men with hypergonadotropic hypogonadism
Koo et al. [19]	USA	Retrospective	41	8.8 (3.7–18.7)	not specified	9.0 (0.6–31.3)	FA	Bu-based regimen	Low dose irradiation	20/41 (48.8%*)	*Calculated in men with hypergonadotropic hypogonadism

Summary of cohort studies assessing the prevalence of gonadal toxicity in men.

The studies are sorted by year of publication. Age and duration of follow-up are given as years with mean (SD), or range where such data are available.

Abbreviations:

Diagnosis: *AA* aplastic anemia, *AML* acute myeloid leukemia, *AUL* acute unclassified leukemia, *ALL* acute lymphoblastic leukemia, *ALD* Adrenoleukodystrophy, *BPDCN* blastic plasmacytoid dendritic cell neoplasm, *β-TM* β-thalassemia major, *CID* congenital immunodeficiency, *CML* Chronic myeloid leukemia, *CLL* Chronic lymphocytic leukemia, *CNS* central nervous system, *FA* Fanconi Anemia, *HB* hepatoblastoma, *HL* Hodgkin’s lymphoma, *JMML* Juvenile Myelomonocytic Leukemia, *MDS* Myelodysplastic syndrome/myelofibrosis, *MM* Multiple myeloma, *NHL* non-Hodgkin’s lymphoma, *OT* Others, *SAA* Severe aplastic anemia/PNH, *SCD* Sickle Cell Disease, *WT* Wilms tumor.

Chemotherapy: *ABVD* doxorubicin, bleomycin, vinblastine and dacarbazine, *ARA-C* cytarabine, *ATG* anti-thymocyte globulin, *BEAM* carmustine, VP16, cytarabine, MEL, *Bu* busulfan, *CDE* doxorubicin, cyclophosphamide, etoposide, and dexamethasone, - *CHOP* cyclophosphamide, doxorubicin, vincristine, and prednisolone, *CsA* cyclosporine A, *Cy* cyclophosphamide, *DHAOX* oxaliplatin, cytarabine, and dexamethasone, *FLUD* fludarabine, *IGEV* ifosphamide, gemcitabine, and vinorelbine, *MEL* melphalan, *MMF* mycophenolate mofetil, *MOPP/ABV(D)* mechlorethamine, vincristine, procarbazine, prednisone, doxorubicin, bleomycin, vinblastine, (dacarbazine), *THIO* thiotepa, *VEPEB* cyclophosphamide, epirubicin, vinorelbine, bleomycin, and prednisolone.

Radiotherapy: *TAI* thoracoabdominal irradiation, *TBI* total body irradiation, *TLI* total lymphoid irradiation.

### Quality assessment

The quality of individual studies was assessed using the Newcastle-Ottawa Scale (NOS) [18]. The three parameters subject selection (0-4 stars), comparability (0-2 stars), and study outcome (0–3 stars) were considered in the scoring of individual studies. The scoring was composed as follows: good quality (= 3 or 4 stars in the selection domain AND 1 or 2 stars in the comparability domain AND 2 or 3 stars in the outcome/exposure domain), fair quality (= 2 stars in the selection domain AND 1 or 2 stars in the comparability domain AND 2 or 3 stars in the outcome/exposure domain), and poor quality (= 0 or 1 star in the selection domain OR 0 stars in the comparability domain OR 0 or 1 stars in the outcome/exposure domain). All of the included studies were reviewed by the AV and the CB to independently assess the risk of bias; disagreements were resolved via consensus. The scoring was conducted according to the terms listed in Table 4.

**Table 4:** Bias screening.

	Selection				Comparability	Outcome					
First author, Year of publication	Representativeness of exposed cohort	Selection of non-exposed cohort	Ascertainment of exposure	Outcome of interest present at study start?	Cohort comparability based on the design or analysis controlled for confounders	Assessment of outcome	Sufficient length of follow-up for outcomes to occur	Adequacy of follow-up of cohorts	Total	Quality Assessment	Comments
Afify et al. [39]	⋆	-	⋆	⋆	-	⋆	⋆	⋆	6/8	Poor	No comparison group
Bakker et al. [43]	⋆	-	⋆	⋆	-	⋆	⋆	⋆	6/8	Poor	No comparison group
Chatterjee et al. [66]	⋆	-	⋆	⋆	-	⋆	⋆	⋆	6/8	Poor	No comparison group
Schmimmer et al. [67]	⋆	-	⋆	⋆	-	⋆	⋆	⋆	6/8	Poor	No comparison group
Anserini et al. [71]	⋆	-	⋆	⋆	-	⋆	⋆	⋆	6/8	Poor	No comparison group
Maeda et al. [106]	⋆	-	⋆	⋆	-	⋆	⋆	⋆	6/8	Poor	No comparison group
Tauchmanova et al. [23]	⋆	⋆	⋆	⋆	⋆	⋆	⋆	⋆	8/8	**Good**	
Li et al. [73]	⋆	-	⋆	⋆	-	⋆	⋆	⋆	6/8	Poor	No comparison group
Somali et al. [74]	⋆	-	⋆	⋆	-	⋆	⋆	⋆	6/8	Poor	No comparison group
Vlachopapadopoulou et al. [75]	⋆	-	⋆	⋆	-	⋆	⋆	⋆	6/8	Poor	No comparison group
Claessens et al. [76]	⋆	-	⋆	⋆	-	⋆	⋆	⋆	6/8	Poor	No comparison group
Couto-Silva et al. [77]	⋆	-	⋆	⋆	-	⋆	⋆	⋆	6/8	Poor	No comparison group
Shalitin et al. [78]	⋆	-	⋆	⋆	-	⋆	⋆	⋆	6/8	Poor	No comparison group
Brachet et al. [35]	⋆	-	⋆	⋆	-	⋆	⋆	⋆	6/8	Poor	No comparison group
Lie Fong et al. [44]	⋆	⋆	⋆	⋆	⋆	⋆	⋆	⋆	8/8	**Good**	
Steffens et al. [40]	⋆	-	⋆	⋆	-	⋆	⋆	⋆	6/8	Poor	No comparison group
Lukusa et al. [42]	⋆	-	⋆	⋆	-	⋆	⋆	⋆	6/8	Poor	No comparison group
Abou-Mourad et al. [86]	⋆	-	⋆	⋆	-	⋆	⋆	-	5/8	Poor	No comparison group
Nabhan et al. [79]	⋆	-	⋆	⋆	-	⋆	⋆	⋆	6/8	Poor	No comparison group
Cho et al. [80]	⋆	-	⋆	⋆	-	⋆	⋆	⋆	6/8	Poor	No comparison group
Felicetti et al. [22]	⋆	-	⋆	⋆	-	⋆	⋆	⋆	6/8	Poor	No comparison group
Ho et al. [81]	⋆	-	⋆	⋆	-	⋆	⋆	⋆	6/8	Poor	No comparison group
Laporte et al. [61]	⋆	-	⋆	⋆	-	⋆	⋆	⋆	6/8	Poor	No comparison group
Borgmann-Staudt et al. [31]	⋆	⋆	⋆	⋆	⋆	⋆	⋆	-	8/8	**Good**	
Khalil et al. [28]	⋆	-	⋆	⋆	-	⋆	⋆	⋆	6/8	Poor	No comparison group
Blijdorp et al. [32]	⋆	⋆	⋆	⋆	⋆	⋆	⋆	⋆	8/8	**Good**	
Inagaki et al. [36]	⋆	-	⋆	⋆	-	⋆	⋆	⋆	6/8	Poor	No comparison group
Krawczuk-Rybak et al. [33]	⋆	⋆	⋆	⋆	⋆	⋆	⋆	⋆	8/8	**Good**	
Poomthavorn et al. [82]	⋆	-	⋆	⋆	-	⋆	⋆	⋆	6/8	Poor	No comparison group
Bresters et al. [51]	⋆	-	⋆	⋆	-	⋆	⋆	⋆	6/8	Poor	No comparison group
Pup et al. [83]	⋆	-	⋆	⋆	-	⋆	⋆	⋆	6/8	Poor	No comparison group
Vatanen et al. [26]	⋆	-	⋆	⋆	-	⋆	⋆	⋆	6/8	Poor	No comparison group
Wilhelmsson et al. [26]	⋆	-	⋆	⋆	-	⋆	⋆	⋆	6/8	Poor	No comparison group
Elchuri et al. [37]	⋆	-	⋆	⋆	-	⋆	⋆	⋆	6/8	Poor	No comparison group
Pfitzer et al. [107]	⋆	-	⋆	⋆	-	⋆	⋆	⋆	6/8	Poor	No comparison group
Barnum et al. [84]	⋆	⋆	⋆	⋆	⋆	⋆	⋆	⋆	8/8	**Good**	
Kawano et al. [52]	⋆	-	⋆	⋆	-	⋆	⋆	⋆	6/8	Poor	No comparison group
Nakano et al. [85]	⋆	-	⋆	⋆	-	⋆	⋆	⋆	6/8	Poor	No comparison group
Vaezi et al. [70]	⋆	-	⋆	⋆	-	⋆	⋆	⋆	6/8	Poor	No comparison group
Gokcebay et al. [46]	⋆	-	⋆	⋆	-	⋆	⋆	⋆	6/8	Poor	No comparison group
Rahal et al. [24]	⋆	-	⋆	⋆	-	⋆	⋆	⋆	6/8	Poor	No comparison group
Ashizawa et al. [87]	⋆	-	⋆	⋆	-	⋆	⋆	⋆	6/8	Poor	No comparison group
Duca et al. [38]	⋆	-	⋆	⋆	-	⋆	⋆	⋆	6/8	Poor	No comparison group
Faraci et al. [88]	⋆	-	⋆	⋆	-	⋆	⋆	⋆	6/8	Poor	No comparison group
Komori et al. [89]	⋆	-	⋆	⋆	-	⋆	⋆	⋆	6/8	Poor	No comparison group
Taneja et al. [105]	⋆	-	⋆	⋆	-	⋆	⋆	⋆	6/8	Poor	No comparison group
Weinhard et al. [90]	⋆	-	⋆	⋆	-	⋆	⋆	⋆	6/8	Poor	No comparison group
Zhao et al. [34]	⋆	-	⋆	⋆	-	⋆	⋆	⋆	6/8	Poor	No comparison group
Bhatt et al. [91]	⋆	-	⋆	⋆	-	⋆	⋆	⋆	6/8	Poor	No comparison group
Boga et al. [92]	⋆	-	⋆	⋆	-	⋆	⋆	⋆	6/8	Poor	No comparison group
Elchuri et al. [23]	⋆	-	⋆	⋆	-	⋆	⋆	⋆	6/8	Poor	No comparison group
Felicetti et al. [21]	⋆	-	⋆	⋆	-	⋆	⋆	⋆	6/8	Poor	No comparison group
Mathiesen et al. [30]	⋆	-	⋆	⋆	-	⋆	⋆	⋆	6/8	Poor	No comparison group
Rostami et al. [104]	⋆	-	⋆	⋆	-	⋆	⋆	⋆	6/8	Poor	No comparison group
Shimazaki et al. [93]	⋆	-	⋆	⋆	-	⋆	⋆	⋆	6/8	Poor	No comparison group
Forgeard et al. [94]	⋆	-	⋆	⋆	-	⋆	⋆	⋆	6/8	Poor	No comparison group
Lopez et al. [103]	⋆	-	⋆	⋆	-	⋆	⋆	⋆	6/8	Poor	No comparison group
Mathiesen et al. [102]	⋆	-	⋆	⋆	-	⋆	⋆	⋆	6/8	Poor	No comparison group
Molinari et al. [95]	⋆	-	⋆	⋆	-	⋆	⋆	⋆	6/8	Poor	No comparison group
Wikander et al. [27]	⋆	-	⋆	⋆	-	⋆	⋆	⋆	6/8	Poor	No comparison group
Xue et al. [96]	⋆	-	⋆	⋆	-	⋆	⋆	⋆	6/8	Poor	No comparison group
Zavattaro et al. [101]	⋆	-	⋆	⋆	-	⋆	⋆	⋆	6/8	Poor	No comparison group
George et al. [97]	⋆	-	⋆	⋆	-	⋆	⋆	⋆	6/8	Poor	No comparison group
Guemes et al. [48]	⋆	-	⋆	⋆	-	⋆	⋆	⋆	6/8	Poor	No comparison group
Su et al. [98]	⋆	-	⋆	⋆	-	-	⋆	⋆	6/8	Poor	No comparison group
Cattoni et al. [100]	⋆	-	⋆	⋆	-	⋆	⋆	⋆	6/8	Poor	No comparison group
Chabut et al. [99]	⋆	-	⋆	⋆	-	⋆	⋆	⋆	6/8	Poor	No comparison group
Jin et al. [20]	⋆	-	⋆	⋆	-	⋆	⋆	⋆	6/8	Poor	No comparison group
Koo et al. [19]	⋆	-	⋆	⋆	-	⋆	⋆	⋆	6/8	Poor	No comparison group

Newcastle-Ottawa Quality Assessment Form for Cohort Studies.

### Data synthesis

The primary outcome of our systematic review was the prevalence of infertility in men and women with malignant or benign haematologic diseases after HSCT treatment. In order to calculate the prevalence, the number of patients who met the criteria for infertility was divided by the number of patients at risk of infertility, as provided by the individual studies. For the pooled prevalence, statistical analyses were performed with the “metafor” function of the R software (R Core Team, Vienna, Austria, 2013). Heterogeneity was examined using Cohen’s Q statistic and the I statistic [2]. In the presence of high heterogeneity, random-effects models were used. To provide clinically meaningful estimates in the meta-analysis, studies with unspecified treatments or < 10 patients were excluded for outcome assessment. However, they were included in the qualitative synthesis and tabular summaries of study characteristics (Table 2).

## Results

### Results of the systematic review

A total of 258 studies were included after screening of the abstracts and full texts. 192 studies were excluded because they did not meet the prespecified inclusion criteria. Finally, 68 articles were included in the systematic review (Fig. 1).

### Study characteristics

The characteristics of the 68 studies are summarized in Tables 2 and 3. The included studies were retrospective (*n* = 57), prospective (*n* = 6), and cross-sectional (*n* = 5). With the exception of five good-quality articles, the majority (*n* = 63) were rated as being of poor methodological quality. This was mainly due to the lack of a comparison group or small sample sizes (Tables 2 and 3).

A total of 4315 patients reported a history of benign/malignant haematologic disease and underwent HSCT, of which 2139 (49.5%) women and 2176 (50.4%) men were eligible for fertility analysis. Study sample sizes ranged from 5 to 217 patients. The studies were conducted in various regions, including Europe (*n* = 39), Asia (*n* = 18), the U.S. (*n* = 7), Canada (*n* = 3) and Australia (*n* = 1).

Benign haematologic diseases included Fanconi Anemia (FA), sickle cell disease (SCD), severe aplastic anemia (SAA), beta-thalassemia major (β-TM), congenital immunodeficiencies (CID), adrenoleukodystrophy (ALD), Blackfan Diamond anemia, Wiskott-Aldrich Syndrome, Glanzmann Syndome, X‐linked lymphoproliferative disease and others (OT). The histology of malignant haematologic diseases included chronic myeloid leukemia (CML), acute myeloid leukaemia (AML), non-Hodgkin’s lymphoma (NHL), Hodgkin’s lymphoma (HL), myelodysplastic syndrome/myelofibrosis (MDS), multiple myeloma (MM), acute lymphoblastic leukaemia (ALL), chronic lymphocytic leukemia (CLL), acute unclassified leukemia (AUL), blastic plasmacytoid dendritic cell neoplasm (BPDCN), Wilms tumour (WT), juvenile myelomonocytic leukemia (JMML), neuroblastoma, nephroblastoma, hepatoblastoma, neuroepithelioma, Ewing sarcoma and others (OT), including rare entities.

Study participants comprised of both prepubertal and post-pubertal males and females, with a median age of 10.8 years (range 0–66) at the time of cancer diagnosis and 22 years (range 1.1–62.4) at the time of outcome evaluation. The studies generally had follow-up periods, with a median of 7.2 years and a range of 0.2–31.3 years. Treatment options included various chemotherapy conditioning protocols with stem cell transplantation and/or different doses and types of radiotherapy. The exact proportion of patients with each specific type of treatment could not be determined.

### Prevalence of infertility

The prevalence of infertility in patients with a history of HSCT ranged from 15% to 100% in women, and from 0% to 100% in men. Retrospective studies of long-term survivors [19–31] (mean follow-up 11 years) with reported infertility prevalence at 61% and 51%, respectively.

### Results of the meta-analysis

Twelve studies that assessed fertility outcomes in fewer than 10 patients ([25, 32–42]) were excluded to provide clinically meaningful estimates (Fig. 1).

### Pooled overall prevalence of infertility after all types of treatment

**Fig. 2:**
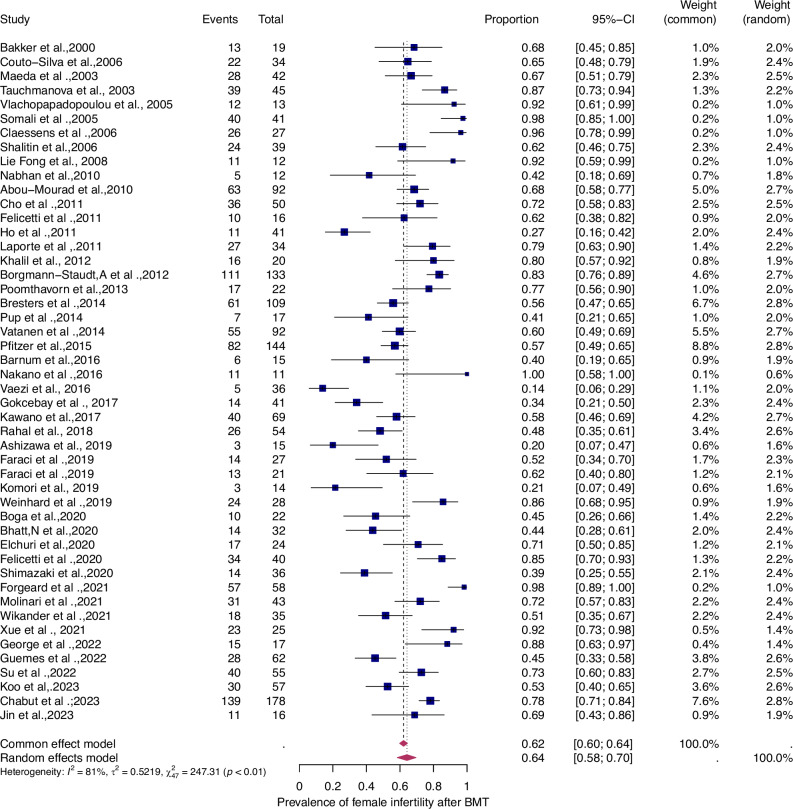
Pooled overall prevalence of gonadal toxicity in women. Forest plot of proportions and 95% confidence intervals (CI) for studies evaluating the prevalence of gonadal toxicity in women following HSCT therapy. Blue squares for each study indicate the proportion, the size of the boxes indicates the weight of the study, and the horizontal lines indicate the 95% CI. The data in bold and pink diamond represent the pooled prevalence for post-treatment infertility and 95% CI. Overall estimates are shown in the fixed- and random effect models.

**Fig. 3:**
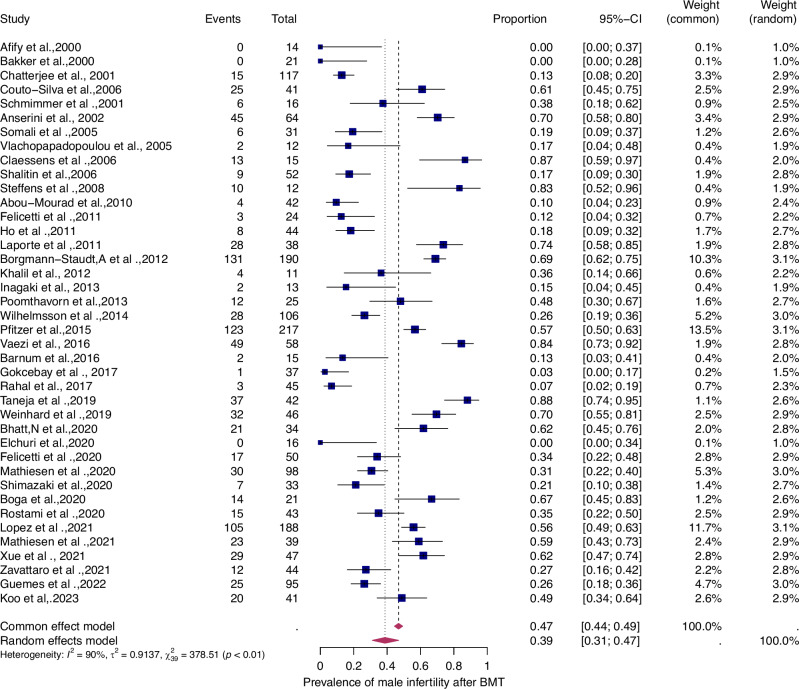
Pooled overall prevalence of gonadal toxicity in men. For details see legend of Fig. 2.

Fifty-six studies were eligible for inclusion in the analysis of the overall prevalence of infertility. These studies comprised 1853 female malignant cases, 241 female benign cases, 1871 male malignant cases, and 226 male benign cases. Consequently, patients were categorized according to their haematological disease, gender, and oncological therapy (i.e. different types and doses of chemotherapy and radiotherapy and combinations of different therapies). The prevalence of each of these studies and a summary of the prevalence are shown in Figs. 2 and 3. The overall prevalence was 64% (95% CI: 58-70%) for women and 39% (31-47%) for men. The heterogeneity test revealed significant heterogeneity among the studies I^2^ = 81, *p* < 0.01 and I^2^ = 90, *p* < 0.01.

### Subgroup analysis: Infertility in patients on the basis of disease type

**Fig. 4:**
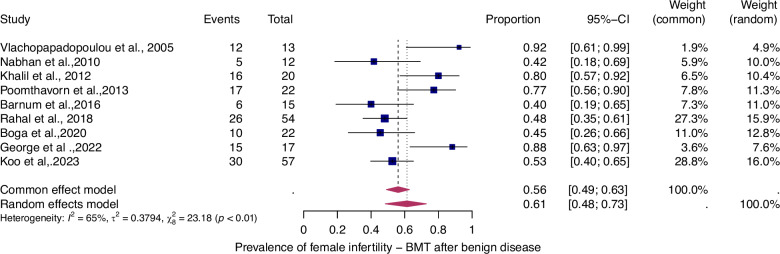
Pooled overall prevalence of gonadal toxicity of benign disease in women. For details see legend of Fig. 2.

**Fig. 5:**
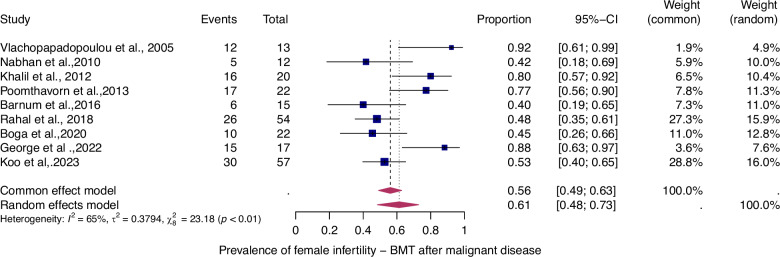
Pooled overall prevalence of gonadal toxicity of malignant disease in women. For details see legend of Fig. 2.

**Fig. 6:**
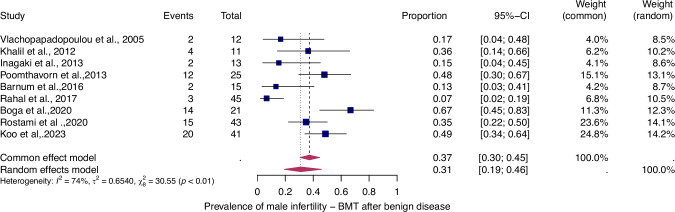
Pooled overall prevalence of gonadal toxicity of benign disease in men. For details see legend of Fig. 2.

**Fig. 7:**
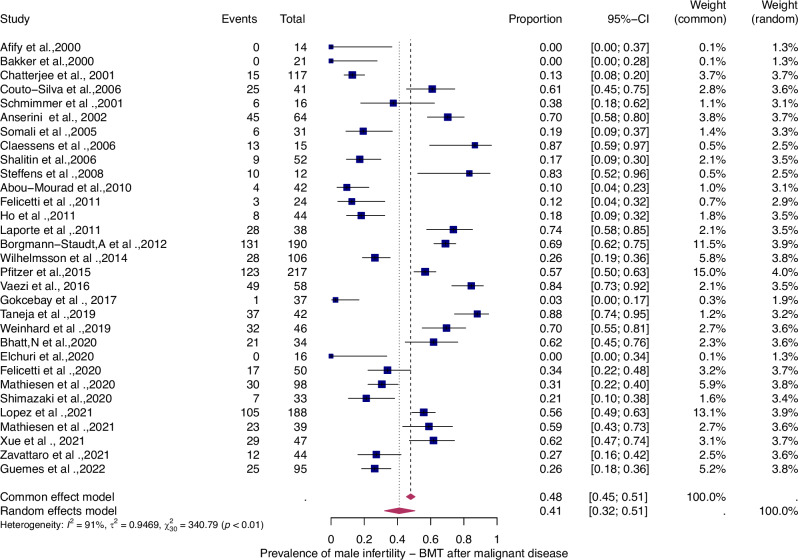
Pooled overall prevalence of gonadal toxicity of malignant disease in men. For details see legend of Fig. 2.

To evaluate the prevalence of infertility as a function of the type of haematologic disease (i.e., benign or malignant), we analyzed four patient groups (Figs. 4–7).

The prevalence of infertility was found to be highest in females with malignant diseases, at 65% (95% CI: 0.58–0.71) (Fig. 5). The prevalence of infertility in females with benign diseases was 61% (95% CI: 0.48–0.73) (Fig. 4). In males, the prevalence of infertility was lower and reached 41% in malignant diseases (95% CI: 0.32–0.51) (Fig. 7) and 31% (95% CI: 0.19–0.46) in benign diseases benign diseases (Fig. 6). Data heterogeneity was high in the female sex, as evidenced in malignant (I^2^ = 83%, *p* < 0.01) and benign (I^2^ = 65%, *p* < 0.01) cases. Also in males, data heterogeneity was high, both in malignant (I^2^ = 91%, *p* < 0.01) and benign (I^2^ = 74%, *p* < 0.01) cases.

## Discussion

The aim of this systematic review and meta-analysis was to analyze the prevalence of gonadotoxicity after HSCT oncological treatment in patients with a history of both benign and malignant haematologic diseases to improve fertility counselling. To the best of our knowledge, it is the first systematic review and meta-analysis of joint prevalence of infertility after a multimodal set of oncological treatments.

Our review revealed the following important findings: First, the overall pooled prevalence of gonadal toxicity in the general population of cancer survivors who had previously undergone HSCT treatment was very high for women (64%; CI 95%: 58–70%) and moderately high for men (39%; CI 95%: 31–47%). Second, we observed a high prevalence of infertility in women both with malignant (65%; 95% CI: 0.58–0.71) and benign diseases (61%; 95% CI: 0.48–0.73).The prevalence of infertility in males with malignant diseases was 41% (95% CI: 0.32–0.51), while the corresponding prevalence in males with benign diseases was 31% (95% CI: 0.19–0.46).

In our review, we discovered six retrospective studies of good quality [31–33, 43–45]. Due to the predominantly mixed therapy study cohorts (i.e., various combinations/doses of chemotherapy and total body irradiation (TBI), aggregated outcomes, and mixed-age populations), it was not possible to perform subgroup analyses of the treatments that occurred in pre-and post-pubertal populations.

Gonadal failure is the most frequent endocrine complication of high-dose chemotherapy and radiotherapy after HSCT; recovery is a rare occurrence [31–33, 43–46]. The risk of hypogonadism increases with patient age at the time of HSCT in both sexes; the younger the age, the better the chance of gonadal recovery [26, 47]. Studies have associated gonadal failure with: older age (> 10 years) at the time of transplantation (in younger individuals there may be gonadal recovery), underlying malignant disease (AML/ALL/lymphomas), second remission of leukemia, cranial/pelvic/total body irradiation, alkylating agents, cisplatin, and nitrosoureas [48]. HSCT is a procedure that is commonly performed in children and young adults. Post-pubertal patients undergoing HSCT have been observed to exhibit higher rates of hypogonadism than their pre-pubertal counterparts. Furthermore, the risk is higher in girls than in boys and in those who receive allogeneic HSCT compared with autologous HSCT [48].

Our data suggest that HSCT is a significant risk factor for infertility in both pre- and post-pubertal females, as well as in males. Notably, women are more affected by infertility than men. In women, the prevalence of infertility remains unchanged regardless of whether the haematological disease is benign or malignant. Nevertheless, there is a slightly lower prevalence of infertility in men with benign haematological disorders than in those with malignant disorders. In the context of benign disease, it is crucial to consider the risk of infertility in both women and men undergoing HSCT in order to provide timely fertility preservation counselling.

Regarding female fertility, according to the Childhood Cancer Survivor Study (CCSS), premature menopause occurs in 8% of survivors and depends on age, ovarian irradiation dose, and cumulative dose of alkylating agents [49]. Alkylating agents impair follicular function, even in reduced-intensity regimens [26, 48–53]. We observed a significant association between BuCy and gonadal toxicity in our systematic review.In a study of adults, Güemes et al. found that women who developed primary hypogonadism were more likely to be treated with busulfan (Bu) for conditioning therapy prior to HSCT (40.6% vs 0%) [48]. Busulfan treatment is associated with higher rates of ovarian dysfunction [39, 48, 54]. A large study by Sanders et al. reported that only 1 in 73 women (1.3%) treated with busulfan/cyclophosphamide (Cy) recovered ovarian function. [55]. Furthermore, busulfan is known to be more gonadally toxic in women than in males [55]. More recently, Vatanen et al. evaluated a group of 92 surviving pubertal women confirming that preserved ovarian function is more frequent in patients conditioned with Cy alone compared to women primed with a regimen containing TBI (29%) or Bu-based (25%) [26]. The incidence of gonadal recovery was between 10% and 13.5% in female patients who received a conditioning regimen containing TBI [55]. Bresters et al. demonstrated that nearly half of girls who were pre- or post-pubertal at the time of HSCT required hormonal induction of puberty and described the association of busulfan with ovarian insufficiency in patients conditioned with chemotherapy alone [51]. In contrast, melphalan has been reported to have the potential to improve ovarian function [39]. Panasiuk et al. reported that girls treated with melphalan combined with fludarabine entered puberty spontaneously and required HRT to a lesser extent compared with girls receiving Bu/Cy [56]. Faraci et al. compared the effect of busulfan and treosulfan (Treo). For female patients, they observed that girls who received Treo during the prepubertal period more frequently reached puberty (menarche) compared to those treated with Bu. This suggests that Treo has a lower impact on pubertal development [25]. For reduced intensity conditioning (RIC), high rates of ovarian function preservation with melphalan-based reduced intensity conditioning have been reported [57]. However, this study neither measured Anti-Mullerian hormone (AMH), semen analyses, nor other parameters of fertility assessment, only follicle stimulation hormone (FSH) [57]. High-dose cyclophosphamide monotherapy is less gonadal toxic than BuCy, especially in young women [58].

Given the potential of RIC as a viable alternative for fertility preservation in HSCT, further studies and the possibility of establishing such a therapy should be considered 1.

Recovery from ovarian insufficiency has been previously described [51]. The prevalence of hypogonadism is higher in older adults [48], and the likelihood of recovery of gonadal function decreases by 20% with each additional year of age [59]. Some studies suggest that AMH may be a useful marker of ovarian follicular reserve in survivors of childhood cancer and/or HSCT [44, 60, 61]. However, although low AMH may be predictive of impending POI, pregnancies were reported to have occurred in women with low AMH values [62].

Regarding male fertility, Hypogonadism affects approximately 15% of young male cancer survivors aged 25–45 years [63]. However, the reported prevalence in studies of adults after allogeneic and autologous HCT varies widely, ranging from 6.9% to 84%. Key factors influencing prevalence include age at transplant, type of conditioning regimen, underlying diseases and previous treatments [64]. Leydig cells, which produce testosterone, are less sensitive to chemotherapy and radiation than the germinal epithelium, where sperm are produced. Recovery from hypogonadism is possible and can sometimes occur within the first year after transplantation [65–70]. However, before reaching a conclusion regarding a patient’s fertility status, it is essential to observe them for a longer period of time, potentially up to 9-10 years [71].

Although the gonadotoxicity of specific chemotherapies can be estimated using risk calculators (www.oncofertilityrisk.com), the assessment should be provided also for benign diseases requiring gonadotoxic treatment. As for the cyclophosphamide Equivalent Dose (CED) Calculator [72], it offers information about the risk of infertility but does not address the availability of multiple treatment options in the same way as other tools do.

There are some limitations to our study, although we strictly followed the recommendations to produce high quality evidence summaries: Firstly, most of the included studies were based on retrospective or registry data and lacked data on treatment protocols or dosimetry of chemotherapy combination and dosage, as well as combination with TBI as an important risk factor for long-term fertility outcomes. Secondly, the heterogeneity of the included studies, due to treatment variations and the diverse characteristics of the study populations, with wide age ranges, did not allow for further subgroup analyses depending on pubertal status, which is of great relevance for pre-treatment fertility protection measure counseling. Thirdly, the absence of data on the dosage of chemotherapy (in mg/m^2^ or CED) precluded the possibility of performing a sub-analysis of CED to assess the aggressiveness of the therapy. A standard conversion to m^2^ was not performed, in order to avoid bias in the results [72].

In conclusion, this first review and meta-analysis assessed the pooled prevalence of suspected infertility after HSCT in malignant and non-malignant haematologic disease. It provides clinically relevant information for fertility prognosis and patient counselling. Given the high prevalence of infertility and the associated risk of long-term complications, fertility preservation methods should be recommended prior to HSCT.

## Supplementary information

S1 Search

## Data Availability

All the data utilized in the study are publicly available and/or contained within the manuscript or appendix.
