# Supplementary material for: Impact of haematopoietic stem cell transplantation for benign and malignant haematologic and non-haematologic disorders on fertility: a systematic review and meta-analysis
Source: Bone Marrow Transplant. 2025 Feb 26;60(5):645–72. doi: 10.1038/s41409-025-02520-6 (PMC12061765; doi:10.1038/s41409-025-02520-6)
Supplement: Supplementary file 1 — S1 Search [file 41409_2025_2520_MOESM1_ESM.docx]

**Table S1** *Database Search Strategies*

Systematic literature search in Medline, Embase and Cochrane CENTRAL

**DATABASE SEARCH STRATEGIES**

Impact of hematopoietic stem cell transplantation for benign and malignant hematologic disorders on fertility: A systematic review and meta-analysis

SEARCH DATE: November 1, 2023

| **Search date** | **Database searched** | **Platform** | **Dates of coverage** | **Records** | **Deduplicated Records** |
| --- | --- | --- | --- | --- | --- |
| 01 Nov 2024 | Embase | Ovid | 1974 – 31 Oct 2023 | 1548 | 1117 |
| 01 Nov 2024 | Medline ALL | Ovid | 1971 – 31 Oct 2023 | 495 | 495 |
| 02 May 2024 | Cochrane Central Register of Controlled Trials | Wiley | 1992 - present | 56 | 32 |
|  |  |  | **Sum of references** | 2099 | **1635** |

**Embase/Ovid**

Search date: 01/11/2023

Embase <1974 to 2023 October 31>

1 exp leukemia/ or exp myelodysplastic syndrome/ or exp acute lymphoblastic leukemia/ or chronic myeloid leukemia/ or exp nonhodgkin lymphoma/ or exp chronic lymphatic leukemia/ or exp Fanconi anemia/ or exp multiple myeloma/ or exp juvenile myelomonocytic leukemia/ or exp chronic myeloid leukemia/ or exp sickle cell anemia/ or exp amegakaryocytic thrombocytopenia/ or exp wiskott aldrich syndrome/ or exp Hodgkin disease/ or exp severe congenital neutropenia/ or exp chronic granulomatous disease/ or exp IPEX syndrome/ or exp myelofibrosis/ or exp POEMS syndrome/ 736043

2 (h?ematolog* or leuk?emi* or leuc?emi* or aml or cll or cml or blast cell cris* or bone marrow displas* or mds or myelodysplas* or refractor* cytopenia or refractor* anemia 5q-syndrome* or jmml or immunocytom* or immunoproliferative small intestinal disease* or lymphoblastom* or lymphosarcoma* or Waldenstroem* or lymphoma* or Burkitt* or hodgkin* or non-hodgkin* or (malign* adj2 (lymphogranuloma* or granuloma*)) or "Reed-sternberg cell*" or "Sternberg-reed cell*" or "Classic* HD" or "classic* HL" or Reticulolymphosarcom* or germinoblastom* or (an?emi* adj2 (Fanconi* or familial hypoplastic or familial aplastic or hereditary hypolastic)) or congenital pancytop?eni* or Fanconi pancytop?eni* or Multiple myelom* or morbus Kahler or Kahler disease or myelom* multiplex or myelomatos* or plasma cell myelom* or (Sickle cell* adj2 (disease* or cris* or an?emia*)) or h?emoglobin sc disease* or h?emoglobin sd disease* or thalassemi* or drepanocyt* or h?emoglobin ss or meniscocytos* or amegakaryocytic thrombocytopeni* or wiskott Aldrich).ti,ab. 1056639

3 1 or 2 1206375

4 (exp bone marrow transplantation/ or exp stem cell transplantation/) and (ae or to).fs. 30811

5 (((bone marrow* or h?ematopoiet* or stem cell* or hsc) adj4 (transplant* or transfer* or graft* or transfus* or infus*or therap*)) or hsct).ti,ab. 205783

6 4 or 5 220124

7 (fertili#ation* or fertility or fertile or fecund* or subfecund* or sub-fecund* or infecund* or infertility or (ovar* adj4 sterility) or subfert* or sub-fert* or anovularit* or gonad* or reproductive organ* or reproduction* or gamete-producing gland* or ovarian reserve* or ovary or ovaries or ovarian follicle* or oogenesis or oocyte* or amenorrhea* or premature menopaus* or early menopaus* or climacterium pr?ecox or Gonadotropin* or AMH or Anti-Mu?llerian Hormone* or Antimu?llerian Hormone* or Anti-Mu?llerian Factor* or Mu?llerian Inhibiting Hormone* or mu?llerian inhibitor* or FSH or Follicle Stimulating Hormone* or Folliculostimulating Hormone* or Follitropin or FSH-releasing hormone* or LH-FSH or testis or testes or testicle* or spermatogenes* or sperm* or semen or gametogenes* or hypogonadism* or hypo-gonadism* or "reproductive system*" or azoospermia* or spermatozoon* or ((resumpt* or recover*) adj3 menstruat*) or menarch* or "menstruat* onset*" or "first menstruat*" or "puberal h?emorrhage*" or (?estrogen* adj3 (substitut* or administrat* or replace* or therap*)) or "hormon* replacement therap*" or hrt or inhibin b or inhibin subunit b).ti,ab. 909617

8 exp fertility/ or exp infertility/ or exp semen analysis/ or exp gonad/ or exp amenorrhea/ or exp early menopause/ or reproduction/ or gametogenesis/ or spermatozoon/ or exp menarche/ or exp estrogen therapy/ or exp inhibin B/ or exp testis size/ 616344

9 7 or 8 1077469

10 3 and 6 and 9 2155

11 (exp animal/ or exp invertebrate/ or nonhuman/ or animal experiment/ or animal tissue/ or animal model/ or exp plant/ or exp fungus/ or (rat? or mouse or mice or animal?).ti.) not (exp human/ or human tissue/) 7817173

12 10 not 11 2086

13 (Editorial or Letter or Note or Review or Book or Chapter or Press).pt. 6373710

14 12 not 13 1675

15 limit 14 to yr="2000 -Current" 1548

*****************************

**Medline/Ovid**

Search date: 01/11/2023

Ovid MEDLINE(R) ALL <1946 to October 31, 2023>

1 exp Leukemia/ or exp Myelodysplastic Syndromes/ or exp hodgkin disease/ or exp lymphoma, non-hodgkin/ or exp Anemia, Hypoplastic, Congenital/ or exp Multiple Myeloma/ or exp Anemia, Sickle Cell/ or exp Wiskott-Aldrich Syndrome/ or exp Granulomatous Disease, Chronic/ or exp Primary Myelofibrosis/ or exp POEMS Syndrome/ 481334

2 (h?ematolog* or leuk?emi* or leuc?emi* or aml or cll or cml or blast cell cris* or bone marrow displas* or mds or myelodysplas* or refractor* cytopenia or refractor* anemia 5q-syndrome* or jmml or immunocytom* or immunoproliferative small intestinal disease* or lymphoblastom* or lymphosarcoma* or Waldenstroem* or lymphoma* or Burkitt* or hodgkin* or non-hodgkin* or (malign* adj2 (lymphogranuloma* or granuloma*)) or "Reed-sternberg cell*" or "Sternberg-reed cell*" or "Classic* HD" or "classic* HL" or Reticulolymphosarcom* or germinoblastom* or (an?emi* adj2 (Fanconi* or familial hypoplastic or familial aplastic or hereditary hypolastic)) or congenital pancytop?eni* or Fanconi pancytop?eni* or Multiple myelom* or morbus Kahler or Kahler disease or myelom* multiplex or myelomatos* or plasma cell myelom* or (Sickle cell* adj2 (disease* or cris* or an?emia*)) or h?emoglobin sc disease* or h?emoglobin sd disease* or thalassemi* or drepanocyt* or h?emoglobin ss or meniscocytos* or amegakaryocytic thrombocytopeni* or wiskott Aldrich).ti,ab. 725726

3 1 or 2 830005

4 exp Bone Marrow Transplantation/ or exp Stem Cell Transplantation/ 139895

5 (((bone marrow* or h?ematopoiet* or stem cell* or hsc) adj4 (transplant* or transfer* or graft* or transfus* or infus*or therap*)) or hsct).ti,ab. 118803

6 4 or 5 178897

7 exp Fertility/ or exp Infertility/ or exp Gonads/ or Amenorrhea/ or anovulation/ or menopause, premature/ or Reproduction/ or Gametogenesis/ or Spermatozoa/ or exp Menarche/ or exp Estrogen Replacement Therapy/ or exp Inhibin-beta Subunits/ 399701

8 (fertili#ation* or fertility or fertile or fecund* or subfecund* or sub-fecund* or infecund* or infertility or (ovar* adj4 sterility) or subfert* or sub-fert* or anovularit* or gonad* or reproductive organ* or reproduction* or gamete-producing gland* or ovarian reserve* or ovary or ovaries or ovarian follicle* or oogenesis or oocyte* or amenorrhea* or premature menopaus* or early menopaus* or climacterium pr?ecox or Gonadotropin* or AMH or Anti-Mu?llerian Hormone* or Antimu?llerian Hormone* or Anti-Mu?llerian Factor* or Mu?llerian Inhibiting Hormone* or mu?llerian inhibitor* or FSH or Follicle Stimulating Hormone* or Folliculostimulating Hormone* or Follitropin or FSH-releasing hormone* or LH-FSH or testis or testes or testicle* or spermatogenes* or sperm* or semen or gametogenes* or hypogonadism* or hypo-gonadism* or "reproductive system*" or azoospermia* or spermatozoon* or ((resumpt* or recover*) adj3 menstruat*) or menarch* or "menstruat* onset*" or "first menstruat*" or "puberal h?emorrhage*" or (?estrogen* adj3 (substitut* or administrat* or replace* or therap*)) or "hormon* replacement therap*" or hrt or inhibin b or inhibin subunit b).ti,ab. 766258

9 7 or 8 883457

10 3 and 6 and 9 845

11 (exp animal/ or exp invertebrate/ or animal experiment/ or animal model/ or exp plant/ or exp fungus/ or (rat? or mouse or mice or animal?).ti.) not exp human/ 5740265

12 10 not 11 814

13 (comment or editorial or letter or meta analysis or "review" or "systematic review").pt. 5563721

14 12 not 13 631

15 limit 14 to yr="2000 -Current" 495

*****************************

**Cochrane Library Trials/Wiley**

Search date: 01/11/2023

#1 [mh Leukemia] OR [mh "Myelodysplastic Syndromes"] OR [mh "hodgkin disease"] OR [mh "lymphoma, non-hodgkin"] OR [mh "Anemia, Hypoplastic, Congenital"] OR [mh "Multiple Myeloma"] OR [mh "Anemia, Sickle Cell"] OR [mh "Wiskott-Aldrich Syndrome"] OR [mh "Granulomatous Disease, Chronic"] OR [mh "Primary Myelofibrosis"] OR [mh "POEMS Syndrome"]

#2 (h?ematolog* OR leuk?emi* OR leuc?emi* OR aml OR cll OR cml OR (blast NEXT cell NEXT cris*) OR (bone NEXT marrow NEXT displas*) OR mds OR myelodysplas* OR (refractor* NEXT cytopenia) OR (refractor* NEXT anemia NEXT 5q-syndrome*) OR jmml OR immunocytom* OR (immunoproliferative NEXT small NEXT intestinal NEXT disease*) OR lymphoblastom* OR lymphosarcoma* OR Waldenstroem* OR lymphoma* OR Burkitt* OR hodgkin* OR non-hodgkin* OR (malign* NEAR/2 (lymphogranuloma* OR granuloma* )) OR ("Reed-sternberg" NEXT cell*) OR ("Sternberg-reed" NEXT cell*) OR (Classic* NEXT "HD") OR (classic* NEXT "HL") OR Reticulolymphosarcom* OR germinoblastom* OR (an?emi* NEAR/2 (Fanconi* OR "familial hypoplastic" OR "familial aplastic" OR "hereditary hypolastic")) OR (congenital NEXT pancytop?eni*) OR (Fanconi NEXT pancytop?eni*) OR (Multiple NEXT myelom*) OR "morbus Kahler" OR "Kahler disease" OR (myelom* NEXT multiplex) OR myelomatos* OR (plasma NEXT cell NEXT myelom*) OR ((Sickle NEXT cell*) NEAR/2 (disease* OR cris* OR an?emia* )) OR (h?emoglobin NEXT "sc" NEXT disease*) OR (h?emoglobin NEXT "sd" NEXT disease*) OR thalassemi* OR drepanocyt* OR (h?emoglobin NEXT "ss") OR meniscocytos* OR (amegakaryocytic NEXT thrombocytopeni*) OR "wiskott Aldrich"):ti,ab

#3 #1 OR #2

#4 [mh "Bone Marrow Transplantation"] OR [mh "Stem Cell Transplantation"]

#5 (((bone NEXT marrow*) OR h?ematopoiet* OR (stem NEXT cell*)):ti,ab OR hsc:ti,ab) NEAR/4 ((transplant* OR transfer* OR graft* OR transfus*):ti,ab OR (infus*or NEXT therap*):ti,ab) OR hsct:ti,ab

#6 #4 OR #5

#7 [mh Fertility] OR [mh Infertility] OR [mh Gonads] OR [mh ^Amenorrhea] OR [mh ^anovulation] OR [mh ^"menopause, premature"] OR [mh ^Reproduction] OR [mh ^Gametogenesis] OR [mh ^Spermatozoa] OR [mh Menarche] OR [mh "Estrogen Replacement Therapy"] OR [mh "Inhibin-beta Subunits"]

#8 (fertili*ation* OR fertility OR fertile OR fecund* OR subfecund* OR sub-fecund* OR infecund* OR infertility OR (ovar* NEAR/4 sterility) OR subfert* OR sub-fert* OR anovularit* OR gonad* OR (reproductive NEXT organ*) OR reproduction* OR (gamete-producing NEXT gland*) OR (ovarian NEXT reserve*) OR ovary OR ovaries OR (ovarian NEXT follicle*) OR oogenesis OR oocyte* OR amenorrhea* OR (premature NEXT menopaus*) OR (early NEXT menopaus*) OR (climacterium NEXT pr*ecox) OR Gonadotropin* OR AMH OR (Anti-Mu*llerian NEXT Hormone*) OR (Antimu*llerian NEXT Hormone*) OR (Anti-Mu*llerian NEXT Factor*) OR (Mu*llerian NEXT Inhibiting NEXT Hormone*) OR (mu*llerian NEXT inhibitor*) OR FSH OR (Follicle NEXT Stimulating NEXT Hormone*) OR (Folliculostimulating NEXT hormone*) OR Follitropin OR (FSH-releasing hormone*) OR LH-FSH OR testis OR testes OR testicle* OR spermatogenes* OR sperm* OR semen OR gametogenes* OR hypogonadism* OR hypo-gonadism* OR (reproductive NEXT system*) OR azoospermia* OR spermatozoon* OR ((resumpt* OR recover*) NEAR/3 menstruat*) OR menarch* OR (menstruat* NEXT onset*) OR (first NEXT menstruat*) OR (puberal NEXT h*emorrhage*) OR (*estrogen* NEAR/3 (substitut* OR administrat* OR replace* OR therap*)) OR (hormon* NEXT replacement NEXT therap*) OR hrt OR (inhibin NEXT b) OR (inhibin NEXT subunit NEXT b)):ti,ab

#9 #7 OR #8

#10 #3 AND #6 AND #9

#11 ([mh animal] OR [mh invertebrate] OR [mh ^"animal experiment"] OR [mh ^"animal model"] OR [mh plant] OR [mh fungus] OR (rat?:ti OR mouse:ti OR mice:ti OR animal?:ti)) NOT [mh human]

#12 #10 NOT #11 with Cochrane Library publication date Between Jan 2000 and Nov 2023, in Trials

Results: 56
